# NLRP1-dependent activation of Gasdermin D in neutrophils controls cutaneous leishmaniasis

**DOI:** 10.1371/journal.ppat.1012527

**Published:** 2024-09-09

**Authors:** Michiel Goris, Katiuska Passelli, Sanam Peyvandi, Miriam Díaz-Varela, Oaklyne Billion, Borja Prat-Luri, Benjamin Demarco, Chantal Desponds, Manon Termote, Eva Iniguez, Somaditya Dey, Bernard Malissen, Shaden Kamhawi, Benjamin P. Hurrell, Petr Broz, Fabienne Tacchini-Cottier

**Affiliations:** 1 Department of Immunobiology, University of Lausanne, Epalinges, Switzerland; 2 WHO Collaborative Center for Research and Training in Immunology, University of Lausanne, Epalinges, Switzerland; 3 Vector Molecular Biology Section, Laboratory of Malaria and Vector Research, Division of Intramural Research, National Institute of Allergy and Infectious Diseases, National Institutes of Health, Rockville, Maryland, United States of America; 4 Post Graduate Department of Zoology, Barasat Government College, Barasat, West Bengal, India; 5 INSERM, CNRS, Centre D’Immunologie de Marseille-Luminy, Aix-Marseille Université, Marseille, France; 6 Department of Molecular Microbiology and Immunology, Keck School of Medicine, University of Southern California, Los Angeles, California, United States of America; INRS - Institut Armand Frappier, CANADA

## Abstract

Intracellular pathogens that replicate in host myeloid cells have devised ways to inhibit the cell’s killing machinery. Pyroptosis is one of the host strategies used to reduce the pathogen replicating niche and thereby control its expansion. The intracellular *Leishmania* parasites can survive and use neutrophils as a silent entry niche, favoring subsequent parasite dissemination into the host. Here, we show that *Leishmania mexicana* induces NLRP1- and caspase-1-dependent Gasdermin D (GSDMD)-mediated pyroptosis in neutrophils, a process critical to control the parasite-induced pathology. In the absence of GSDMD, we observe an increased number of infected dermal neutrophils two days post-infection. Using adoptive neutrophil transfer in neutropenic mice, we show that pyroptosis contributes to the regulation of the neutrophil niche early after infection. The critical role of neutrophil pyroptosis and its positive influence on the regulation of the disease outcome was further demonstrated following infection of mice with neutrophil-specific deletion of GSDMD. Thus, our study establishes neutrophil pyroptosis as a critical regulator of leishmaniasis pathology.

## Author summary

Leishmaniases are neglected infectious diseases with around 1 million new cases reported per year. Neutrophils are rapidly and massively recruited to the site of infection. Several *Leishmania* species including *Leishmania mexicana*, inhibit the host neutrophil antimicrobial response, using these cells as an entry shelter. Here, we discover a novel host defense mechanism against *Leishmania mexicana* that controls neutrophil presence at the onset of infection. The inflammasomes are multiprotein signaling complexes that detect infection and induce pyroptotic cell death. We show that the NLRP1 inflammasome is important in the control of lesion development and parasite burden. We identify NLRP1-driven GSDMD pyroptosis in neutrophils as critical in the control of the early neutrophil pool, impacting the outcome of infection. Taken together, we show that neutrophil pyroptosis is an essential player in cutaneous leishmaniasis.

## Introduction

The host’s innate immune defense relies on a multitude of pattern-recognition receptors and signaling sensors to initiate an immune response to invading pathogens or cell perturbations. These include NOD-like receptors (NLRs), several of which can form a multiprotein signaling platform called the inflammasome [1]. After the detection of cell perturbations, the NLRs interact with pro-caspase-1, either directly through CARD-CARD interactions or by using the adaptor protein ASC, which leads to procaspase-1 autoproteolysis and activation [2]. In turn, caspase-1 cleaves pro-interleukin (IL)-1β and IL-18 cytokines into their biologically active forms, as well as the pore-forming protein Gasdermin D (GSDMD) [3–5]. GSDMD exists in an autoinhibited state, with an inhibitory C-terminus shielding the cytotoxic N-terminal domain (GSDMD-NT). Upon cleavage in the linker region by caspase-1, GSDMD-NT inserts into the cell membrane to form a pore, which acts as a conduit for IL-1β and IL-18 release and aids in the antimicrobial proinflammatory response. In addition, a non-canonical pathway can be activated, mediated by murine caspase-11 (caspase-4/5 in humans), which directly cleaves GSDMD to activate the NLRP3 inflammasome through potassium efflux [6]. If enough GSDMD pores form, the cell undergoes pyroptosis, a lytic, inflammatory form of cell death that releases danger signals, alerting the environment during an ongoing infection [7].

Pyroptosis is an essential mechanism of defense against pathogens residing in vacuoles and the cytosol as it contributes to the antimicrobial response by eliminating the pathogen’s replication niche [8]. Pyroptosis can be triggered upon recognition of different pathogens by various inflammasomes. The mechanisms leading to pyroptosis have been well characterized in macrophages, however, those involved in neutrophils have been less investigated. Early studies showed that neutrophils can resist caspase-1-induced pyroptosis [9,10]. However, recent work reported pore formation and neutrophil pyroptosis upon caspase-1 activation [11,12], suggesting that the outcome of caspase-1 activation and subsequent pyroptosis in neutrophils depends on the stimulus and the type of inflammasome involved [13].

Leishmaniases are a group of neglected diseases that include diverse clinical forms, ranging from cutaneous skin lesions, the most prevalent form, to mucocutaneous and visceral forms, the latter being fatal if not treated [14]. *Leishmania* are intracellular protozoan parasites, which are recognized by several sensors, including Toll-like receptors and inflammasomes [15,16]. Following infection, activation of the NLRP3 inflammasome was shown to have either a protective or a detrimental impact on the disease pathology, depending on the infecting *Leishmania* species (spp.) [17–20]. In addition to NLRP3, higher transcript levels of AIM-2 and NLRP1 have been reported in localized skin lesions of patients with cutaneous leishmaniasis due to *L*. *braziliensis* and *L*. *amazonensis* [21]. *Toxoplasma gondii*, another protozoan parasite, was also shown to activate the NLRP1 inflammasome [22], suggesting that this inflammasome may also play a role in *Leishmania* infection.

*Leishmania* parasites are transmitted through the bite of an infected female sand fly, which deposits infectious metacyclic promastigotes during blood feeding. Skin damage and vector-derived factors egested during the infectious blood meal induce a rapid and intense neutrophil recruitment to the site of infection, characterized by chemokine and cytokine release and upregulation of IL-1β [17,23–27]. Neutrophils are the first line of defense against pathogens [28] and are characterized by their high microbicidal properties including phagocytosis, degranulation, and neutrophil extracellular traps (NETs) formation. Neutrophils can sequester *Leishmania* parasites through phagocytosis and the production of NETs [29,30], however, most *Leishmania* spp. causing cutaneous leishmaniasis have developed strategies to resist neutrophil microbicidal action [31,32]. These *Leishmania* spp. use neutrophils as a Trojan horse to silently enter macrophages, their final host cell, and induce an anti-inflammatory, permissive host for the parasites, promoting their further growth [33,34]. The mechanisms involved in the deleterious role of neutrophils at the onset of infection may vary depending on the infecting *Leishmania* spp. Neutrophils play a deleterious role after *L*. *mexicana* infection [27] thus, in this study, we investigated whether *L*. *mexicana* could induce neutrophil pyroptosis, thereby modulating the extent of their contribution to pathology. Furthermore, we explored the upstream inflammasome events leading to neutrophil pyroptosis.

Here we show that *L*. *mexicana* infection involves the NLRP1 inflammasome, which induces caspase-1-dependent GSDMD activation in neutrophils, regulating the magnitude of the pathology. Using mice genetically deficient for GSDMD in neutrophils and performing adoptive neutrophil transfers in neutropenic mice, we show that neutrophil pyroptosis reduces the early parasite neutrophil niche, restricting parasite load and its dissemination at the onset of infection, with long-term impact on the disease. These findings confirm the crucial role of neutrophils during *L*. *mexicana* infection and show that GSDMD-induced pyroptosis is a protective mechanism in this pathology. This study expands the understanding of inflammasome activation and pyroptosis in neutrophils, opening new therapeutic targets for *Leishmania* and other diseases in which neutrophils play a crucial role.

## Results

### ASC-dependent inflammasome signaling does not influence the outcome of *L*. *mexicana* infection

**Fig 1:**
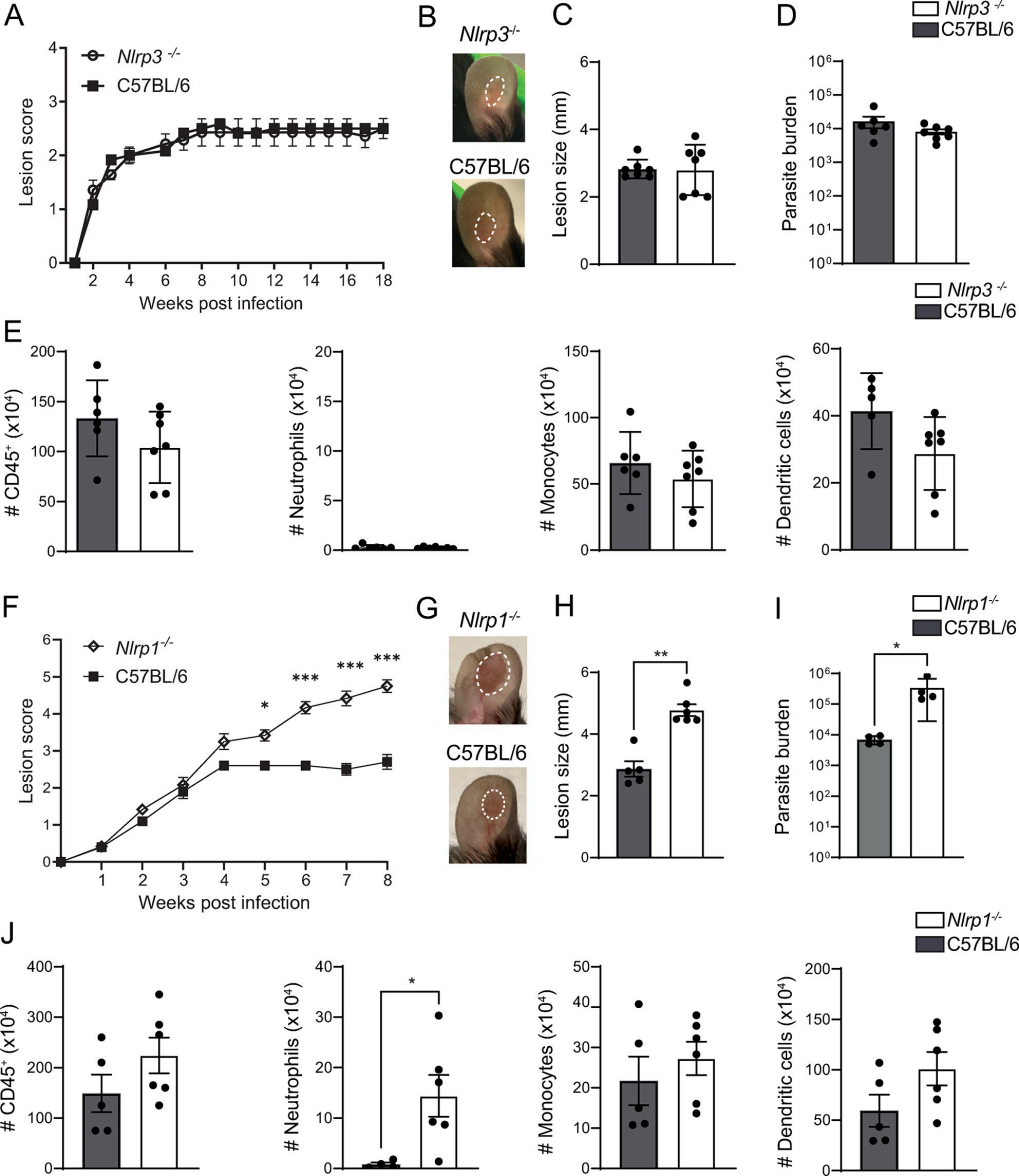
NLRP1, but not NLRP3, protects against lesion exacerbation during *L*. ***mexicana* infection. (A)** *Nlrp3*^-/-^ and C57BL/6 control mice were infected i.d. with 10^6^ *L*. *mexicana* metacyclic promastigotes. Lesion development was measured weekly over the indicated time frame, and lesion score was determined including lesion size, inflammation, and pathology status. **(B)** Eighteen weeks p.i., representative lesion pictures are shown, **(C)** lesion size was measured and **(D)** parasite load at the site of infection was determined by limiting dilution assay (LDA). **(E)** The number of CD45^**+**^ cells and the relevant immune populations, including CD45^**+**^CD11b^+^Ly6G^+^ neutrophils, CD45^**+**^CD11b^+^Ly6C^+^ monocytes, and CD45^**+**^CD11c^+^ dendritic cells in the infected ear, was determined by flow cytometry. **(F)** *Nlrp1*^*-/-*^ and C57BL/6 control mice were similarly infected i.d. with metacyclic *L*. *mexicana* promastigotes and lesion score was monitored over 8 weeks. **(G)** Representative pictures of *Nlrp1*^*-/-*^ and C57BL/6 ear lesions at 8 weeks p.i. **(H)** Representative ear lesion size and **(I)** parasite load as determined by LDA. **(J)** The number of CD45^**+**^ cells, CD45^+^CD11b^+^Ly6G^+^ neutrophils, CD45^+^CD11b^+^Ly6C^+^ monocytes, and CD45^+^CD11c^+^ dendritic cells in the infected ears was analyzed 8 weeks p.i. by flow cytometry. Data are shown as mean ± SD and are representative of ≥3 experiments, n≥4/group. Statistical differences in lesion development were analyzed by 2-way ANOVA, and cell numbers using a Mann-Whitney U-test. *p <0.05; **p <0.01. ***p <0.001.

The NLRP3 inflammasome activation has been reported to have a protective or detrimental role during infection with different *Leishmania* spp., depending on the spp. or strain [16,35]. To determine whether NLRP3 activation has an impact on *L*. *mexicana* pathology, we infected *Nlrp3*^*-/-*^ and C57BL/6 wild-type (WT) control mice intradermally (i.d.) with 10^6^ *L*. *mexicana* metacyclic promastigotes and observed lesion development by assessing the score, which includes the inflammation as well as the length and width of the lesion [36]. Interestingly, no significant difference in lesion score was observed 5 weeks post infection (p.i.), when differences between susceptible and resistant mice usually appear [37]. No difference between both genotypes was observed up to 18 weeks p.i., with both groups exhibiting similar chronic, non-progressive lesion development, with no significant differences in lesion score, lesion size (Fig 1A–1C), and in parasite load at the infection site as determined by limited dilution analysis (LDA) (Fig 1D). At the end of the experiment, immune cell infiltration at the site of infection, including neutrophils, monocytes, and dendritic cells was analyzed by flow cytometry as shown in the gating strategy (S1A Fig). The number and frequency of cells was similar between the two genotypes (Figs 1E and S1B) except for a small difference in dendritic cell frequency that was not statistically significant in other independent experiments. These data indicate that the NLRP3 inflammasome does not play a significant role in *L*. *mexicana-*induced pathology.

Non-canonical inflammasome activation through the direct recognition of *Leishmania* by caspase-11 was previously reported for *L*. *amazonensis* [19]. We thus assessed whether *L*. *mexicana* infection could also activate caspase-11. We infected *Casp-11*^*-/-*^ mice with *L*. *mexicana* and compared lesion evolution and pathology with that of C57BL/6 mice. Similar lesion development and parasite burden were observed in both groups (S2A–S2C Fig). At the end of the experiment, there was no difference in the number and frequency of CD45^+^ cells (S2D Fig) and in neutrophil, monocyte, and dendritic cell number between *Casp-11*^*-/-*^ and C57BL/6 mice (S2E Fig). These data show that *L*. *mexicana*-induced pathology is not influenced by non-canonical inflammasome activation.

NLRP3, as well as other inflammasome sensors lacking a CARD domain, rely on the adaptor protein ASC for the recruitment of caspase-1. We thus assessed if other ASC-dependent inflammasomes could be triggered by *L*. *mexicana*. To this end, we infected *Asc*^*-/-*^ mice i.d., and measured lesion development for several weeks. *Asc*^*-/-*^ mice displayed a comparable lesion score and size to C57BL/6 controls (S2F–S2G Fig). Both genotypes showed similar parasite burden at the site of infection (S2H Fig). Additionally, the number and frequency of CD45^+^ cells, including neutrophils, monocytes, and dendritic cells present was similar between both genotypes as analyzed by flow cytometry (S2I and S2J Fig). These findings suggest a negligible role of ASC-dependent inflammasomes during *L*. *mexicana* infection.

The NLRC4 inflammasome can function in absence of ASC, through direct interaction of its CARD domain with the caspase-1 CARD domain [38]. To assess a putative role of this inflammasome, *Nlrc4*^*-/-*^ mice were infected with *L*. *mexicana*, and disease development was evaluated. Mice genetically deficient for the NLRC4 inflammasome developed similar lesion score, size, and parasite burden as control C57BL/6 mice (S2K–S2M Fig). As previously observed in infected *Nlrp3*^*-/-*^ and *Asc*^*-/-*^ mice, no difference in the number and frequency of immune cell infiltration at the site of infection was detected compared to control C57BL/6 mice (S2N and S2O Fig). Together, these results rule out a role for the activation of classical ASC-dependent inflammasomes, the non-canonical inflammasome, and NLRC4 during *L*. *mexicana* infection.

### The NLRP1 inflammasome prevents *L*. *mexicana-*induced lesion exacerbation in mice

The NLRP1 inflammasome contains a CARD domain and can bypass the absolute requirement of ASC to activate caspase-1 [39,40]. Moreover, NLRP1 activation by *Toxoplasma gondii*, another protozoan parasite, has been reported [22,41]. In C57BL/6 mice, three tandem paralogs (*Nlrp1a*, *Nlrp1b* and *Nlrp1c*) encode for NLRP1. We thus infected *Nlrp1*^*-/-*^ mice, which are deficient in all three forms, i.d. with *L*. *mexicana* and measured lesion development. Five weeks post-infection, *Nlrp1*^*-/-*^ mice developed a significantly larger lesion score than control C57BL/6 mice (Fig 1F). This difference increased thereafter, as shown in representative pictures and lesion size at 8 weeks p.i. (Fig 1G and 1H). *Nlrp1*^*-/-*^ mice displayed significantly higher parasite burden at the site of infection compared to C57BL/6 mice (Fig 1I). Of interest, there was a significant increase in neutrophil number at the lesion site of *Nlrp1*^*-/-*^ mice compared to control mice, with similar number and frequency of CD45^+^ cells, monocytes and dendritic cells (Figs 1J and S3A).

To explore the potential impact of NLRP1 on adaptive immunity, we analyzed by flow cytometry the T helper cytokine profile in the draining lymph nodes (dLNs) and infected ears 8 weeks p.i. No difference was observed in the frequency of CD4^+^ IFN-γ^+^ T cells, CD8^+^ IFN-γ^+^ T cells or CD4^+^ IL-4^+^ T cells in dLNs and at the infection site between *Nlrp1*^*-/-*^ mice and control mice (S3B and S3C Fig). Collectively, these data show that NLRP1 protects against lesion exacerbation and contributes to parasite control but does not influence the development of an adaptive immune response.

### Caspase-1 and Gasdermin D have a protective role during *L*. *mexicana* infection

**Fig 2:**
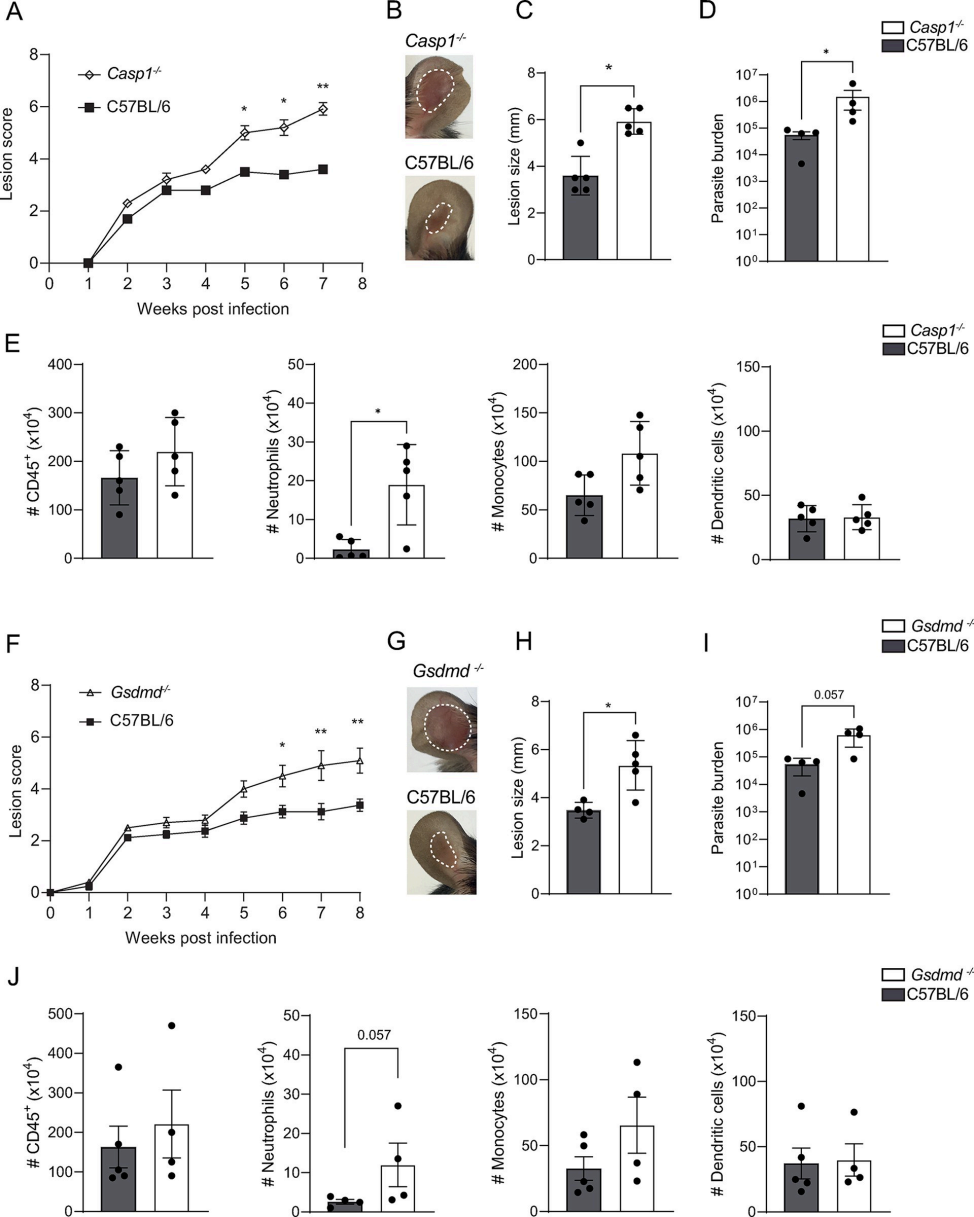
Caspase-1 and Gasdermin D (GSDMD) reduce *L*. *mexicana-*induced pathology and parasite burden. **(A)** *Casp1*^-/-^and C57BL/6 control mice were infected i.d. with metacyclic *L*. *mexicana* promastigotes and lesion score was measured over time. **(B)** Representative pictures of ear lesion, **(C)** ear lesion size and **(D)** parasite load determined by LDA at the site of infection. **(E)** The number of CD45^**+**^ immune cells, CD45^+^CD11b^+^Ly6G^+^ neutrophils, CD45^+^CD11b^+^Ly6C^+^ monocytes, and CD45^+^CD11c^+^ dendritic cells at the infection site, was analyzed by flow cytometry **(F)** *Gsdmd*^-/-^ and C57BL/6 control mice were similarly infected with *L*. *mexicana* and lesion score assessed over time. **(G)** Representative pictures of ear lesions **(H)** lesion size and **(I)** parasite load determined by LDA at 8 weeks p.i. **(J)** Total number of CD45^**+**^ cells, CD45^+^CD11b^+^Ly6G^+^ neutrophils, CD45^+^CD11b^+^Ly6C^+^ monocytes, and CD45^+^CD11c^+^ dendritic cells at the infection site. Data are representative of ≥3 experiments, n≥4/group. Statistical differences in lesion development were analyzed with a 2-way ANOVA with repeated measures, and cell numbers using a Mann-Whitney U-test. *p <0.05; **p <0.01.

NLRP1 activation causes downstream caspase-1 clustering and autoproteolytic activation. To determine if the observed phenotype was due to a classical NLRP1-Caspase-1 axis, we infected mice genetically deficient in caspase-1 (*Casp-1*^*-/-*^) with *L*. *mexicana*. After 5 weeks of infection, *Casp1*^*-/-*^ mice exhibited a significantly higher lesion score, lesion size, and parasite burden than control C57BL/6 mice, and this difference continued to increase until 7 weeks p.i. (Fig 2A–2D). The number and frequency of CD45^+^ cells were slightly increased with a neutrophil number detected at the site of infection that was significantly more elevated in *Casp1*^*-/-*^ mice compared to control mice, while that of monocytes, and dendritic cells was not significantly increased (Figs 2E and S4A). At the same timepoint, caspase-1 did not impact the development of the adaptive immune response, as no difference was observed in the frequency of CD4^+^IFN-γ^+^ T cells, CD8^+^IFN-γ^+^ T cells and CD4^+^IL-4^+^ T cells in the dLNs and at the site of infection of *Casp1*^*-/-*^ and C57BL/6 control mice (S4B and S4C Fig).

Gasdermin D (GSDMD) is a key substrate for caspase-1 and a central player in pyroptotic cell death. Neutrophil cell death mechanisms, like NETosis, have been shown to involve GSDMD [10]. Additionally, GSDMD has recently been shown to protect against *Leishmania* lesion aggravation [20]. Following long-term infection with *L*. *mexicana*, *Gsdmd*^*-/-*^ mice also displayed exacerbated lesions (Fig 2F–2H) and an increased parasite burden (Fig 2I). Furthermore, a threefold increase in neutrophil number was detected at the site of infection, together with increased CD45^+^cells and neutrophil frequency (Figs 2J and S4D). The frequency of CD4^+^IFN-γ^+^ T cells, CD8^+^IFN-γ^+^ T cells, and CD4^+^IL-4^+^ T cells in the dLNs and at the site of infection of *Gsdmd*^*-/-*^ and C57BL/6 control mice (S4E and S4F Fig) remained unchanged, as determined by flow cytometry. The exacerbated pathology observed following infection of *Gsdmd*^-/-^ mice with a high dose (10^6^ metacyclic promastigote parasites) of *L*. *mexicana* was confirmed using a lower infection dose (10^4^ metacyclic promastigote parasites), which better mimics the inoculum deposited by a sandfly during a natural infection [42]. Similar to our observations following a high-dose infection, the lesion score, size, and the parasite burden were elevated in *Gsdmd*^-/-^ compared to control mice (S5A–S5D Fig).

### *L*. *mexicana* induces neutrophil pyroptosis

GSDMD pores can facilitate the release of IL-1β through the cell membrane. The role of GSDMD in macrophage pyroptosis is well-defined, but its involvement during infection with *Leishmania* has only recently been described [20]. To assess if *L*. *mexicana* infection triggers inflammasome activation in macrophages, we quantified IL-1β release *in vitro* by ELISA. IL-1β release was detected 16 hours p.i. in bone marrow-derived macrophages (BMDMs) in a dose-dependent manner, and this secretion was decreased in the absence of caspase-1 and GSDMD, but not in the absence of NLRP1 (S6 Fig). These findings suggest that *in vitro*, IL-1β release in macrophages is independent of NLRP1. Consequently, the NLRP1-dependent phenotype observed following infection likely results from NLRP1 activation in other cell types.

**Fig 3:**
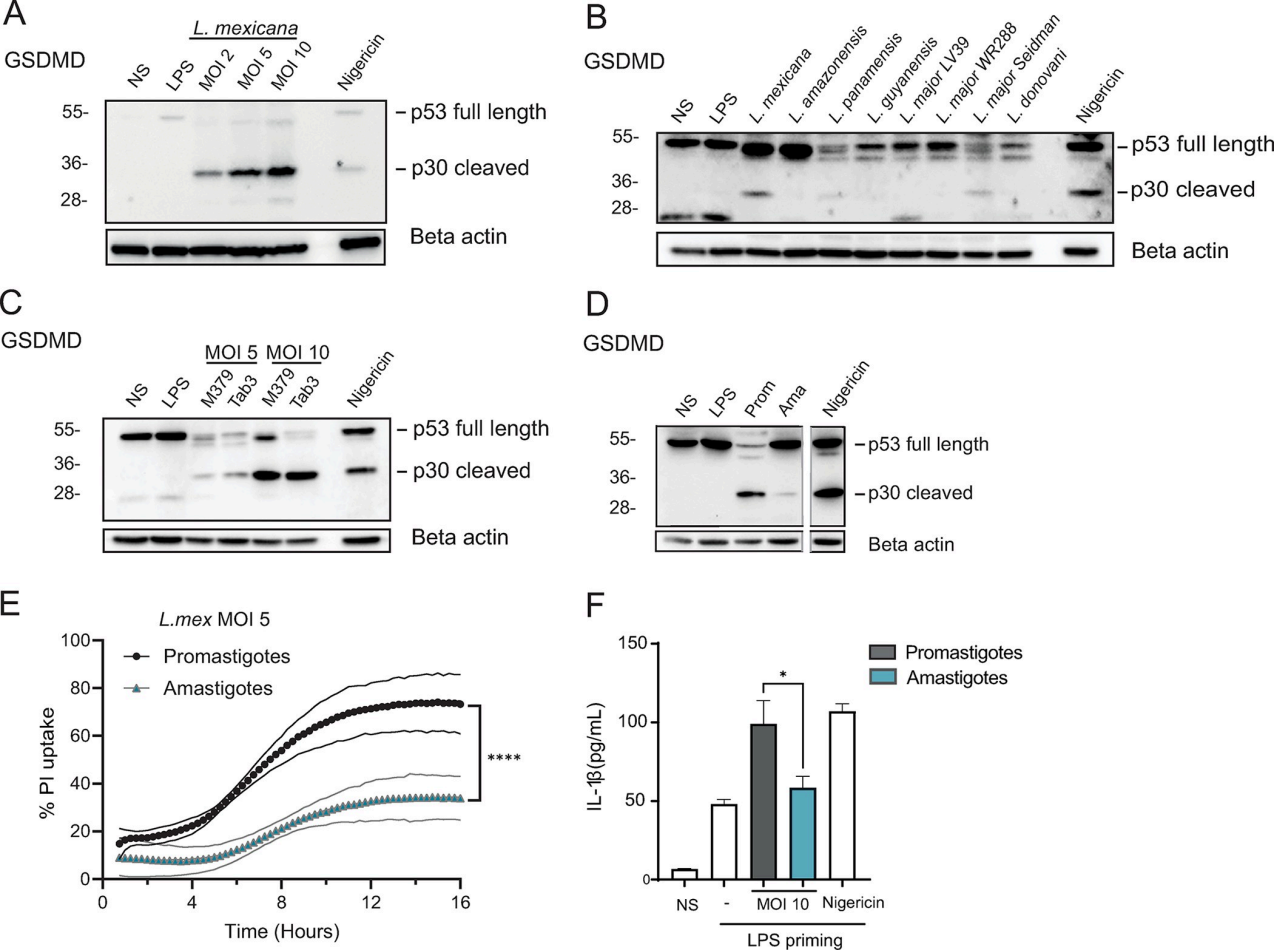
*L*. *mexicana* leads to inflammasome activation in neutrophils. **(A)** Bone marrow-derived C57BL/6 neutrophils (BMNs) were isolated, primed with LPS and infected with *L*. *mexicana* promastigotes at a multiplicity of infection (MOI) of 2, 5, and 10 for 16 hours, or exposed to Nigericin for 4 hours as a positive control. Immunoblotting of GSDMD and beta-actin was performed on cell extracts. **(B)** BMNs were similarly infected with other *Leishmania* species at the MOI 5. **(C)** Comparison of GSDMD cleavage between *L*. *mexicana* M379 and Tab3 strains at the indicated MOI. **(D)** C57BL/6 BMNs were isolated, primed with LPS and infected at a MOI of 10 with *L*. *mexicana* promastigotes or axenic amastigotes, as indicated. Immunoblotting of GSDMD and beta-actin was performed on cell extracts and Nigericin was used as a positive control (same blot and exposure). **(E)** LPS-primed C57BL/6 BMNs were infected with *L*. *mexicana* promastigotes and axenic amastigotes at MOI of 5 and propidium iodide (PI) uptake was quantified over 18 hours of infection. **(F)** The corresponding IL-1β release in supernatants was assessed by ELISA. NS (non-stimulated), LPS (LPS 100ng/ mL priming alone), MOI (multiplicity of infection), Pro (promastigotes), Ama (amastigotes). Data are representative of >2 independent experiments. Differences between populations are analyzed by 2-way ANOVA **(E)** and Unpaired t-test **(F)**.

Neutrophils play a pivotal role during *L*. *mexicana* infection and early neutrophil presence can negatively impact disease progression, preventing the parasite from exploiting the neutrophil niche for lesion establishment [27]. To assess whether *L*. *mexicana* was able to induce inflammasome activation in neutrophils, we infected LPS-primed C57BL/6 bone marrow-derived neutrophils (BMNs) with *L*. *mexicana* promastigotes for 16 hours and analyzed GSDMD activation by Western Blot. We observed a dose-dependent cleavage of full-length GSDMD (p53) into the active p30 N-terminal part in cell lysates, suggesting inflammasome activity in infected neutrophils (Fig 3A). To investigate if GSDMD cleavage is a common process in other *Leishmania* spp., we infected LPS-primed C57BL/6 BMNs for 16 hours with New-World *Leishmania* spp., including *L*. *mexicana*, *L*. *amazonensis*, *L*. *panamensis* and *L*. *guyanensis* and Old-World *Leishmania* spp. and strains, including *L*. *major* LV39, *L*. *major* WR288, *L*. *major* Seidman and *L*. *donovani* (Fig 3B). Infection with *L*. *mexicana* induced GSDMD cleavage, contrary to the other New World spp. tested, except for *L*. *panamensis*, where GSDMD cleavage was observed at a lower level. Among the Old-World spp. tested, GSDMD activation in neutrophils was selectively induced by *L*. *major* Seidman, in line with the reported importance of the NLRP3 inflammasome and neutrophils in the pathology induced by this *L*. *major* strain [17]. Thus, the highest induction of GSDMD cleavage was observed following *L*. *mexicana* infection. To rule out possible variations due to long-time *in vitro* culture, we used the same *L*. *mexicana* strain after demonstrating its vector competency through the development of mature transmissible infections in sand flies (S7A Fig.). Infection with both *L*. *mexicana* strains resulted in similar GSDMD cleavage showing that the phenotype observed did not arise during *in vitro* culture of the parasites (S7B Fig.). We also analyzed GSDMD cleavage in neutrophils infected with another *L*. *mexicana* strain, TAB3, isolated from a patient with diffuse cutaneous lesions [43]. Similar GSDMD cleavage was observed in neutrophils infected with both strains (Fig 3C). In the mammalian host, infectious *Leishmania* promastigotes rapidly transform into amastigotes, its intracellular replicating form. To investigate if *L*. *mexicana* amastigotes would also induce neutrophil pyroptosis, axenic *L*. *mexicana* amastigotes were prepared, and LPS-primed neutrophils were infected at a MOI of 10. In parallel, neutrophils were similarly infected with *L*. *mexicana* promastigotes. We observed GSDMD cleavage following infection with both life stages, although much lower levels of GSDMD cleavage were observed after infection with amastigotes than promastigotes (Fig 3D). As a correlate of cell membrane permeability, uptake of propidium iodide (PI), a nucleic acid dye, was measured in response to promastigotes and axenic amastigotes at multiplicity of infection 5 (MOI 5). Both *L*. *mexicana* life stages induced PI uptake, but at a significantly lower level in response to amastigotes, in line with the lower GSDMD cleavage observed (Fig 3E). Furthermore, infection with amastigotes also induced IL-1β release by neutrophils, again at lower levels than those infected by promastigote forms of the parasites (Fig 3F). Collectively our data reveal that both forms of *L*. *mexicana* induce neutrophil pyroptosis, with a higher induction by the early promastigote stage.

**Fig 4:**
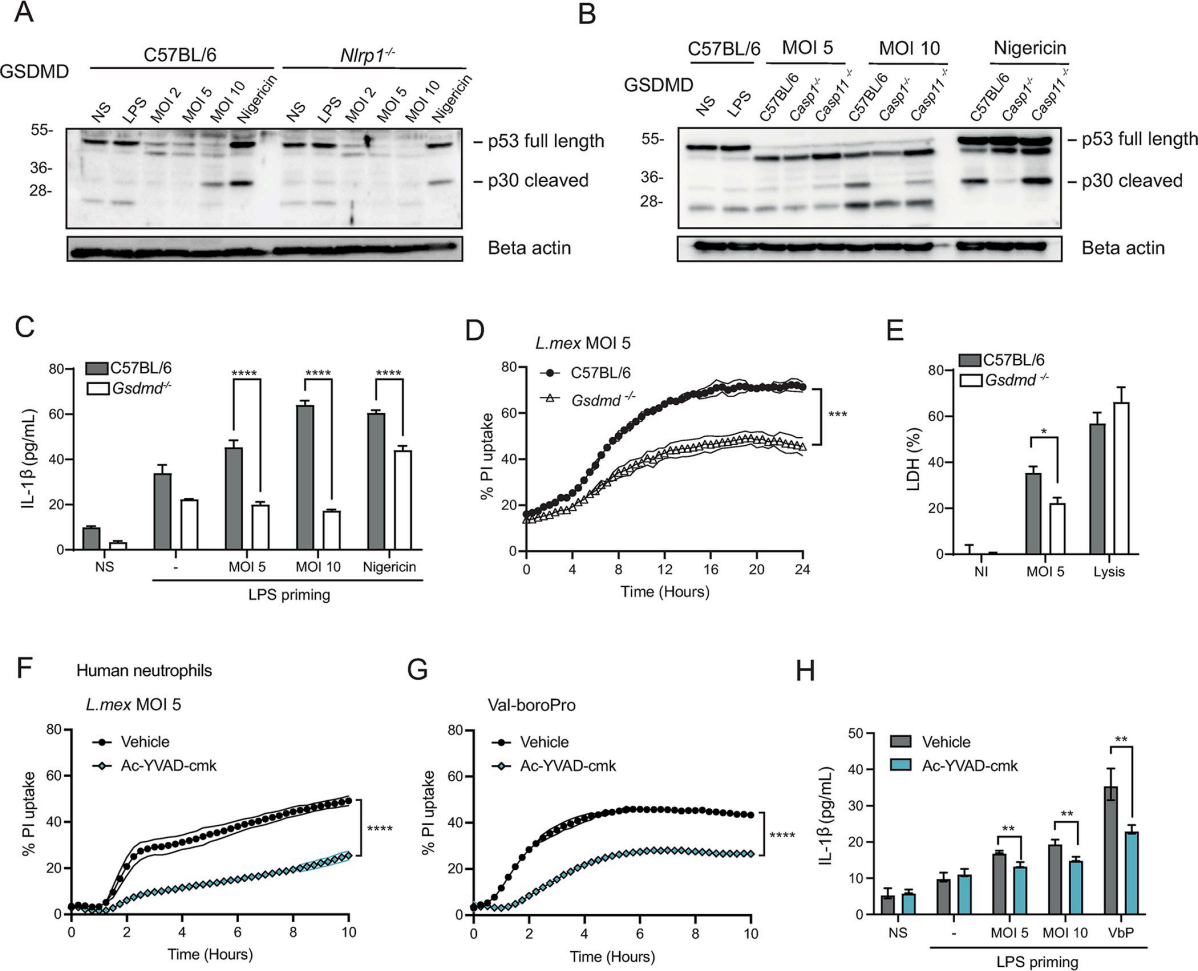
*mexicana* leads to NLRP1-dependent GSDMD-cleavage and pyroptosis in neutrophils. ***L*.** BMNs were primed with LPS and infected *with L*. *mexicana* promastigotes at the indicated MOI for 16 hours or exposed to Nigericin for 4 hours as a positive control. **(A)** Immunoblot of GSDMD and beta-actin of infected *Nlrp1*^*-/-*^ and C57BL/6 BMNs. **(B)** Immunoblot for GSDMD and beta-actin of *Casp1*^-/-^, *Casp11*^*-/-*^ and C57BL/6 BMNs after infection with *L*. *mexicana*. Images are representative of n>3 independent experiments. **(C)** IL-1β release was measured by ELISA in LPS-primed *Gsdmd*^-/-^ and C57BL/6 BMN supernatant after 16 hours of infection. **(D)** LPS-primed *Gsdmd*^*-/-*^ and C57BL/6 BMNs were infected with *L*. *mexicana* promastigotes at MOI of 5 and propidium iodide (PI) uptake was quantified over 24 hours of infection. **(E)** LPS-primed BMNs were infected with *L*. *mexicana* for 16 hours and lactate dehydrogenase (LDH) release was analyzed, with values expressed as a percentage of total LDH upon full lysis of BMNs. Data are shown as mean ± SD and representative of n>3 independent experiments. **(F)** Human neutrophils from healthy donors were primed with LPS and infected with *L*. *mexicana* at MOI of 5 and PI uptake analyzed in presence or absence of the caspase-1 inhibitor ac-YVAD-cmk **(G)**. Human neutrophils were similarly primed and exposed to the NLRP1 activator Val-boroPro in presence or absence of caspase-1 inhibitor. **(H).** IL-1β release was measured in human neutrophils exposed to *L*. *mexicana* at the indicated MOI or to Val-boroPro, in the presence or absence of caspase-1 inhibitor. These results are representative of three experiments. NS (non-stimulated), NI (non-infected), LPS (LPS 100ng/ mL for mouse neutrophils, 500ng/mL for human neutrophils), VbP (Val-boroPro at 10μM). **(D, F, G)** 2-way ANOVA with Tukey’s multiple comparisons test, **(C, H)** Unpaired t-test. *p <0.05; **p <0.01. ***p <0.001, ****p<0.0001.

Our *in vivo* data demonstrated a role for the NLRP1 inflammasome in the control of infection. To investigate whether NLRP1 activation in neutrophils was responsible for *L*. *mexicana*-induced GSDMD cleavage, we infected LPS-primed *Nlrp1*^-/-^ and C57BL/6 BMNs with *L*. *mexicana* for 16 hours and analyzed GSDMD cleavage by Western Blot. A strong reduction of GSDMD cleavage was detected in *Nlrp1*^*-/-*^ neutrophils (Fig 4A), indicating that NLRP1 is upstream of GSDMD activation during neutrophil infection, in line with our *in vivo* results. In addition, we examined if GSDMD cleavage was mediated by caspase-1. We infected LPS-primed *Casp1*^*-/-*^ and *Casp11*^*-/-*^ BMNs and analyzed GSDMD cleavage by Western Blot. We could not detect any cleaved p30 N-terminal GSDMD in *Casp1*^*-/-*^ neutrophils, while GSDMD cleavage was detected in *Casp11*^*-/-*^ neutrophils (Fig 4B). Altogether, these data strongly support the activation of a NLRP1-caspase-1-GSDMD axis in neutrophils upon *L*. *mexicana* infection. LPS-primed BMNs released IL-1β upon *L*. *mexicana* infection 16 hours p.i., a process abrogated in *Gsdmd*^-/-^ neutrophils (Fig 4C). In addition, to assess whether GSDMD activation in neutrophils led to pore formation in infected neutrophils, LPS-primed BMNs were infected with *L*. *mexicana* promastigotes at MOI of 5, and uptake of PI was measured over 24 hours as a correlate of cell permeability. *L*. *mexicana-*induced PI uptake was significantly reduced in *Gsdmd*^*-/-*^ neutrophils compared to C57BL/6 controls (Fig 4D). We then quantified lactate dehydrogenase (LDH) release at 16 hours p.i, to measure if pore formation led to cell lysis and pyroptosis. LDH release was lower in *Gsdmd*^*-/-*^ compared to C57BL/6 infected neutrophils, showing GSDMD-dependent LDH release (Fig 4E). These results indicate that *L*. *mexicana* induces GSDMD-dependent, lytic pyroptosis. Of note, no difference in apoptosis was observed between *L*. *mexicana*-infected *Gsdmd*^*-/-*^ and control neutrophils, as analyzed by Annexin-V / DAPI staining. (S8A and S8B Fig). These findings suggest that infection of neutrophils with *L*. *mexicana* leads to GSDMD cleavage and pyroptosis, with no impact on non-lytic, apoptotic cell death. Collectively our data show that *L*. *mexicana* induces GSDMD activation in neutrophils, leading to pyroptotic cell death.

Human NLRP1 differs from murine NLRP1 as far as the number of paralogs coding for NLRP1 (3 in C57BL/6 mice and 1 in human), requirement of ASC (dispensable in mNLRP1b) and cell expression, with human NLRP1 reported to be mostly expressed in epithelial cells and keratinocytes, while murine NLRP1 is mainly expressed in myeloid cells [44]. To assess if *L*. *mexicana-*also induces pyroptosis in human neutrophils, we isolated peripheral blood neutrophils from healthy human donors and infected them with *L*. *mexicana*. We first assessed inflammasome-dependent cell permeability by measuring PI uptake. Neutrophils were primed with LPS and exposed to *L*. *mexicana*, or to *L*. *mexicana* and the caspase-1 inhibitor ac-YVAD-cmk. PI uptake was observed following infection, a process that was markedly reduced in presence of caspase-1 inhibitor (Fig 4F), revealing inflammasome activation in *L*. *mexicana*-infected human neutrophils. Furthermore, the NLRP1 activator Val-boroPro similarly induced PI uptake in neutrophils, a process that was also reduced upon inhibition of caspase-1 (Fig 4G), suggesting that NLRP1 can also be activated in human neutrophils. In line with these results, induction of IL-1β release by human neutrophils was observed in response to both *L*. *mexicana* or Val-boroPro, and secretion was decreased in presence of the caspase-1 inhibitor (Fig 4H). Collectively, these data reveal that the parasites can trigger pyroptosis in both murine and human neutrophils and suggest that NLRP1 can be triggered in human neutrophils.

GSDMD has been implicated in neutrophil extracellular trap (NET) formation [10,45]. We thus assessed dsDNA release *in vitro* through Picogreen dsDNA detection and detected a small, but statistically significant GSDMD-dependent increase in extracellular dsDNA release in response to *L*. *mexicana* (S9A Fig). To further evaluate the impact of GSDMD on *L*. *mexicana*-induced NET formation, *Gsdmd*^-/-^, *Casp1*^-/-^ and C57BL/6 bone marrow-derived neutrophils and human neutrophils isolated from peripheral blood of healthy donors were infected with *L*. *mexicana*. The formation of NETs was visualized and quantified by confocal microscopy following DAPI and MPO staining. *L*. *mexicana* induced NET formation in neutrophils from all mouse genotypes and in human neutrophils (S9B and S9C Fig). No statistically significant difference was observed in the frequency of NETs between *L*. *mexicana*-infected *Gsdmd*^-/-^, *Casp1*^-/-^ and C57BL/6 neutrophils (S9B Fig). These data suggest that the small decrease observed in dsDNA release in *Gsdmd*^-/-^ neutrophils likely comes from reduced pyroptosis.

GSDMD had no major impact on other neutrophil functions, as we did not observe changes in radical oxygen species (ROS) production levels in *Gsdmd*^*-/-*^ compared to C57BL/6 BMNs 2 hours p.i (S10A and S10B Fig). Furthermore, no impact of GSDMD on *L*. *mexicana* internalization at 2 hours p.i. (S10C Fig) was observed, with a small impact on infection rate observed 24 hours p.i. Additionally, we did not observe an impact of GSDMD on neutrophil activation status (S10D Fig) as compared to C57BL/6 control cells.

### GSDMD regulates the neutrophil niche at the onset of *L*. *mexicana* infection

**Fig 5:**
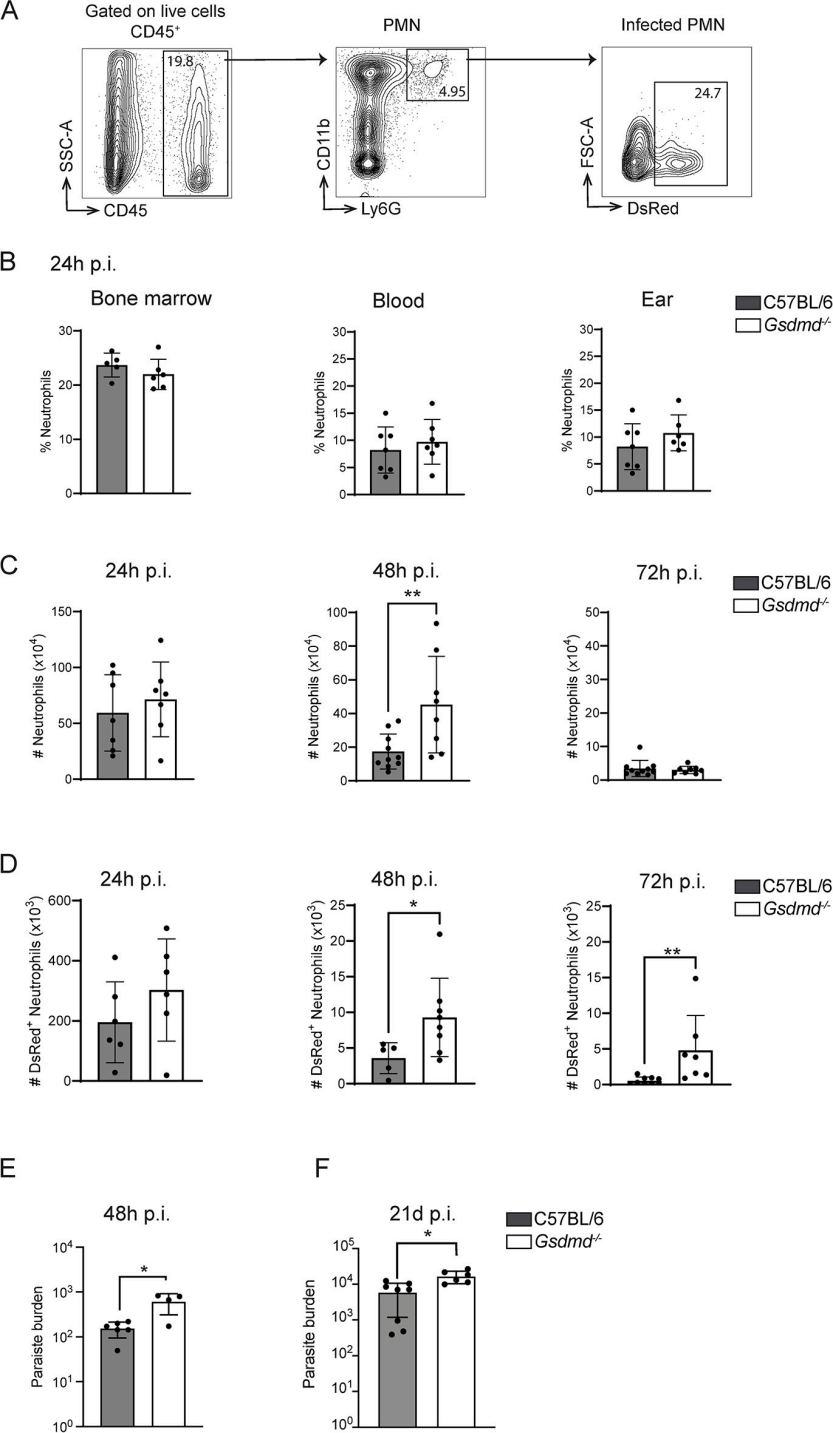
GSDMD regulates the *L*. *mexicana* neutrophil niche during the first days of infection. **(A)** C57BL/6 and *Gsdmd*^*-/-*^ mice were infected i.d with metacyclic dsRed^+^ *L*. *mexicana* promastigotes and the number and frequency of neutrophils in the bone marrow, blood, and at the infection site was determined by flow cytometry at the indicated timepoints. The flow cytometry gating strategy is shown. **(B)** The frequency of CD45^+^CD11b^+^Ly6G^+^ neutrophils at 24 hours p.i. in bone marrow, blood, and infected ear is represented **(C)** The number of CD45^+^CD11b^+^Ly6G^+^ neutrophils present at the infection site at 24, 48, and 72 hours p.i., as analyzed by flow cytometry and **(D)** the number of *L*. *mexicana*-DsRed^+^ CD45^+^CD11b^+^Ly6G^+^ C57BL/6 and *Gsdmd*^-/-^ infected neutrophils is given for each genotype and analyzed time point p.i. **(E)**. The parasite burden in the infected C57BL/6 and *Gsdmd*^*-/-*^ ear was determined by limiting dilution (LDA) analysis 48 hours p.i. **(F)** C57BL/6 and *Gsdmd*^-/-^ mice were similarly infected with *L*. *mexicana* and 21 days p.i, the parasite burden assessed by LDA in infected ears. Data are shown as mean ± SD and are representative of n>2 experiments per time point, with n>4 mice/group. Mann-Whitney U-test *p<0.05, **p<0.01.

*L*. *mexicana* has evolved ways to exploit neutrophils as a niche to establish initial infection and interfere with a protective immune response [27,46]. To assess if neutrophil recruitment was impacted by GSDMD *in vivo*, we monitored neutrophil frequency in the blood, BM, and at the site of infection (Fig 5B). At 24 hours post-*Leishmania* infection, neutrophil frequency in the BM and the blood was similar in C57BL/6 and *Gsdmd*^*-/-*^ mice, indicating that GSDMD has no impact on the initial neutrophil recruitment at this timepoint (Fig 5B). A small but not significant increase in neutrophil frequency was detected at the site of infection (Fig 5B). To determine neutrophil dynamics at the site of infection, we assessed the number of neutrophils at 24, 48 and 72 hours p.i. (Fig 5C). As previously noted, the number of neutrophils was comparable in *Gsdmd*^*-/-*^ and C57BL/6 mice at 24 hours p.i., and decreased in both genotypes at 48 hours p.i., but at a significantly slower rate in *Gsdmd*^*-/-*^ compared to C57BL/6-infected ears. By 72 hours p.i., neutrophil numbers returned to basal levels in both groups (Fig 5C), correlating with the frequency of cells (S11A Fig). Over the same time course, we analyzed the corresponding number of infected neutrophils using dsRed-expressing *L*. *mexicana* parasites. A significantly higher number of infected neutrophils was detected at 48 hours and 72 hours post-infection in *Gsdmd*^*-/-*^ mice compared to the control group (Figs 5D and S11B). This increase in infected neutrophils correlated with an increased parasite burden observed in the infected ear 48 hours post infection (Fig 5E), as determined by limiting dilution analysis (LDA). Altogether, these data show that GSDMD acts as a negative regulator of neutrophil survival during *L*. *mexicana* infection, thereby reducing the neutrophil niche exploited by the parasite.

Following infection with *L*. *mexicana*, neutrophils are rapidly recruited to the site of infection and return to basal levels by 3 days p.i. A second wave of neutrophils arises around 3 weeks post infection [37], a time at which the size of the inflammatory lesion between *Gsdmd*^*-/-*^ and C57BL/6 mice does not differ significantly yet (Figs 2F and 7A), and only the amastigote form is present. The parasite burden measured by LDA in the infected *Gsdmd*^*-/-*^ ears was higher than control C57BL/6 mice (Fig 5F). These data suggest that *L*. *mexicana*-induced pyroptosis also contributes to the regulation of parasite load during the second wave of neutrophil recruitment.

**Fig 6:**
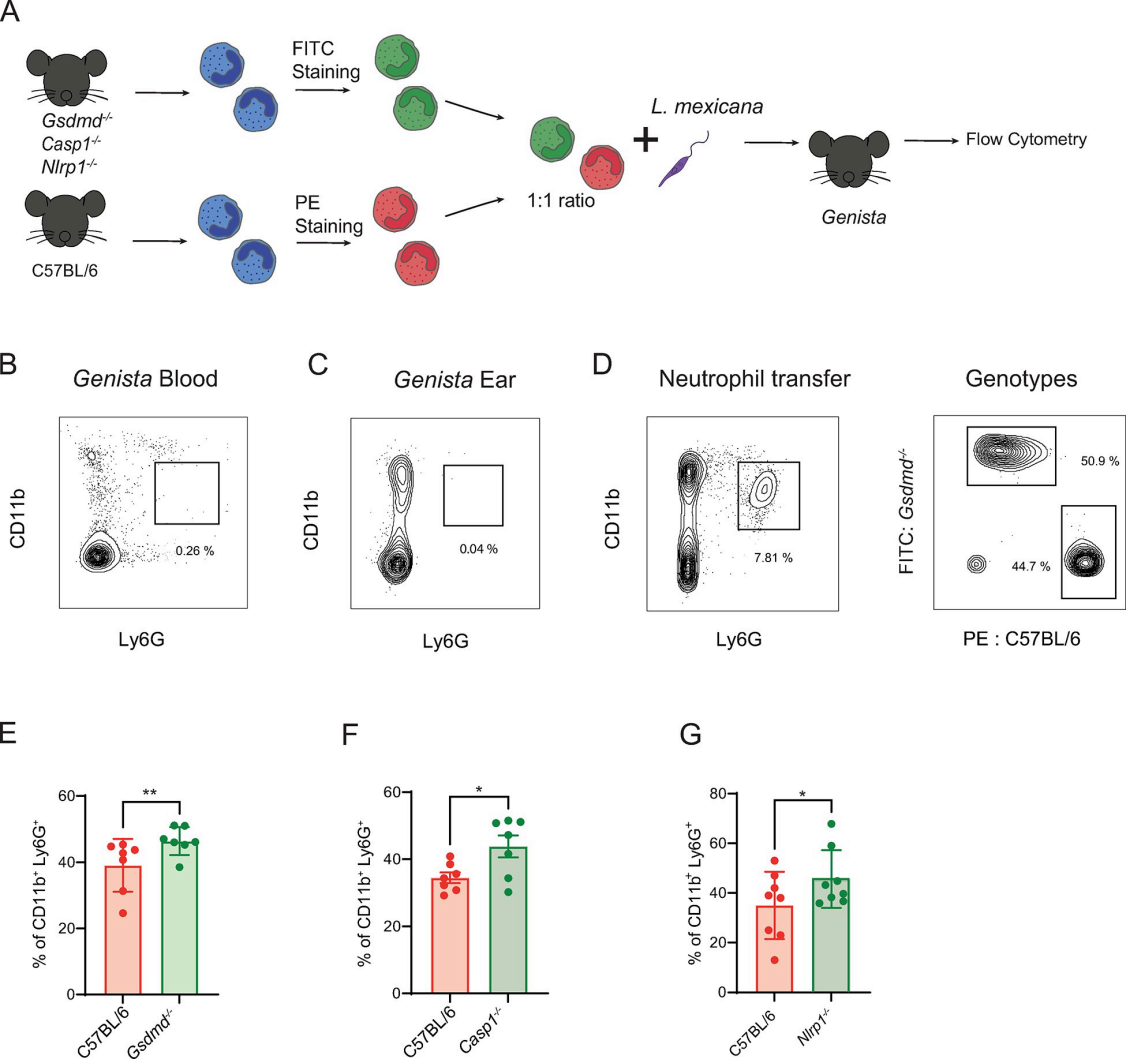
NLRP1 regulates the neutrophil niche through caspase-1 and GSDMD-dependent pyroptosis. **(A)** BMNs isolated from naïve *Gsdmd*^*-/-*^, *Casp1*^*-/-*^, *Nlrp1*^*-/-*^ mice or C57BL/6 control neutrophils were stained with the indicated fluorescent dyes, mixed at a 1:1 ratio and transferred into *Genista* neutropenic mice at the time of infection with *L*. *mexicana* metacyclic promastigotes, as represented in the experimental design. Representative flow cytometry plot showing the lack of mature CD11b^+^Ly6G^+^ neutrophils in **(B)** peripheral blood and (**C**) uninfected ear of *Genista* mice. **(D)** The gating strategy to analyse the genotype of the two transferred neutrophil populations present in *Genista* mice is shown. Bar-plot showing the relative frequency of **(E)** *Gsdmd* ^-/-^ **(F)**, *Casp1*^*-/-*^ **(G)** and *Nlrp1*^*-/-*^ neutrophils compared to the simultaneously transferred C57BL/6 neutrophils at the site of infection 24 hours p.i. Data are shown as mean ± SD and are representative of n>2 experiments, with n>4 ears/group. Wilcoxon signed-rank test, *p<0.05, **p<0.01.

To strengthen these findings, we conducted an adoptive transfer experiment in neutropenic *Genista* mice [47], designed to assess neutrophil survival at the site of infection, in absence of neutrophil recruitment. *Gsdmd*^*-/-*^ and C57BL/6 BMNs were isolated and stained with distinct cell tracker dyes and co-injected with *L*. *mexicana* into the ear dermis of neutropenic *Genista* mice. The frequency of *Gsdmd*^*-/-*^ and C57BL/6 neutrophils was analyzed 24 hours later, by flow cytometry (Fig 6A). *Genista* mice showed no mature neutrophils as expected, in the blood (Fig 6B) and in the ear (Fig 6C). Following adoptive transfer at the time of infection, a higher frequency of *Gsdmd*^*-/-*^ compared to C57BL/6 neutrophils was observed 24h later in the infected ear (Fig 6D and 6E), indicating prolonged survival of GSDMD-deficient neutrophils. As we showed that caspase-1 and NLRP1 are upstream of GSDMD cleavage in *L*. *mexicana*-infected neutrophils *in vitro*, we performed similar adoptive transfer experiments with a mix of C57BL/6 neutrophils and either *Casp1*^*-/-*^ or *Nlrp1*^*-/-*^ neutrophils. Again, a higher frequency of either *Casp1*^*-/-*^ and *Nlrp1*^*-/-*^ neutrophils was observed in the infected ear dermis 24 hours p.i. (Fig 6F and 6G). Together, these results confirm that during *L*. *mexicana* infection *in vivo*, NLRP1-dependent caspase-1 activation of GSDMD, and subsequent pyroptosis control the neutrophil pool at the site of infection.

### Neutrophil-specific expression of GSDMD regulates the *L*. *mexicana-*driven pathology

**Fig 7:**
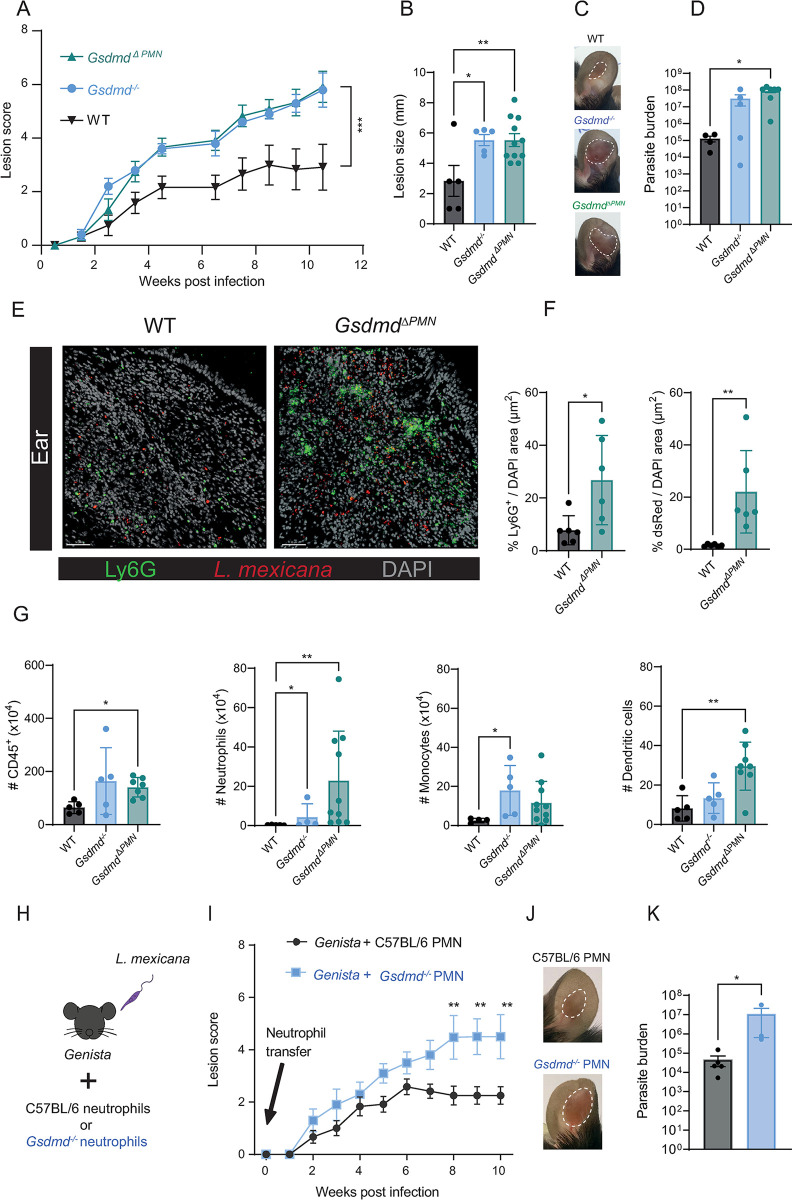
Early GSDMD activation in neutrophils reduces *L*. *mexicana*-driven pathology and parasite burden. **(A)** *Gsdmd*^*-/-*^, *Gsdmd*^ΔPMN^, and WT, mice were infected i.d. with *L*. *mexicana* promastigotes, and lesion development was measured. **(B)** Ear lesion size at 11 weeks p.i. and **(C)** representative pictures of WT, *Gsdmd*^*-/-*^ and *Gsdmd*^ΔPMN^ infected ears. **(D)** Parasite load was determined by LDA. **(E)** Representative images of immunohistology of ear cryosections at 8 weeks p.i., stained for Ly6G^+^ neutrophils (green), dsRed-expressing *L*. *mexicana* parasites (red), and DAPI (grey). Scale bar, 50 μm. **(F)** Quantification of immunofluorescence images. **(G)** Eleven weeks p.i. the number of CD45^**+**^ cells, CD45^+^CD11b^+^Ly6G^+^ neutrophils, CD45^+^CD11b^+^Ly6C^+^ monocytes, and CD45^+^CD11c^+^ dendritic cells at the infection site, was analyzed by flow cytometry **(H)** C57BL/6 and *Gsdmd*^-/-^ BMNs were isolated and adoptively transferred at the time of infection in two separate groups of *Genista* mice as indicated in the experimental design. **(I)** Ear lesion score was measured over the indicated time. **(J)** Ten weeks p.i., representative pictures of the indicated infected ear are shown and **(K)** the number of living parasites was determined by LDA. Data are shown as mean ± SD and are representative of 2 experiments, n≥3/group. Statistical differences in lesion development were analyzed with a 2-way ANOVA repeated measures, and differences in parasite load and cell populations were analyzed with Mann-Whitney U test. *p <0.05; **p <0.01.

To confirm that the increased pathology observed in *Gsdmd*^-/-^ mice was driven by neutrophils, we generated *Mrp8*^*cre*^*Gsdmd*^*lox/lox*^ mice, hereafter referred to as *Gsdmd*^ΔPMN^ mice, that have a neutrophil-specific deletion of GSDMD. We then infected *Gsdmd*^ΔPMN^, *Gsdmd*^*-/-*^ and control *Mrp8*^*WT*^*Gsdmd*^*lox/lox*^ mice with *L*. *mexicana* and compared lesion and pathology development. Both *Gsdmd*^ΔPMN^ and *Gsdmd*^*-/-*^ mice exhibited similar, significantly increased lesion score, size, and exacerbated pathology as compared to control mice (Fig 7A–7C). Similarly, enhanced parasite burden was observed at the site of infection in both *Gsdmd*^ΔPMN^ and *Gsdmd*^*-/-*^ mice, as compared to control mice (Fig 7D). We visualized the higher parasite load observed in *Gsdmd*^ΔPMN^ mice by immunofluorescence of infected ears 8 weeks p.i. and detected a higher density of neutrophils in infected ears (Figs 7E, 7F and S12A). Locally, higher levels of CD45^+^ cells were observed by flow cytometry in both *Gsdmd*^ΔPMN^ and *Gsdmd*^*-/-*^ infected ears (Fig 7G). Additionally, we observed an increase in neutrophil number and frequency, as well as an increase in dendritic cell and monocytes numbers and frequency in *Gsdmd*^ΔPMN^ compared to C57BL/6 infected ears (Figs 7G and S12B). The immune response was similar in the three genotypes with comparable frequency of CD4^+^IFNγ^+^, CD4^+^ IL-4^+^ and CD4^+^IL-17^+^ T cells observed in dLN of cells 11 weeks and 21 days p.i. (S12C and S12D Fig). Furthermore, at 21 days p.i. the levels of arginase and iNOS in dermal macrophages, as detected by flow cytometry of infected ear cells, were similar in the three genotypes (S12E Fig). These data suggest that the increased number of infected neutrophils observed in absence of GSDMD does not result from impaired microbicidal activity of macrophages but from reduced neutrophil pyroptosis. These results highlight the protective role of the NLRP1-caspase-1-GSDMD axis in neutrophils during *L*. *mexicana* infection and subsequent skin pathology.

**Fig 8:**
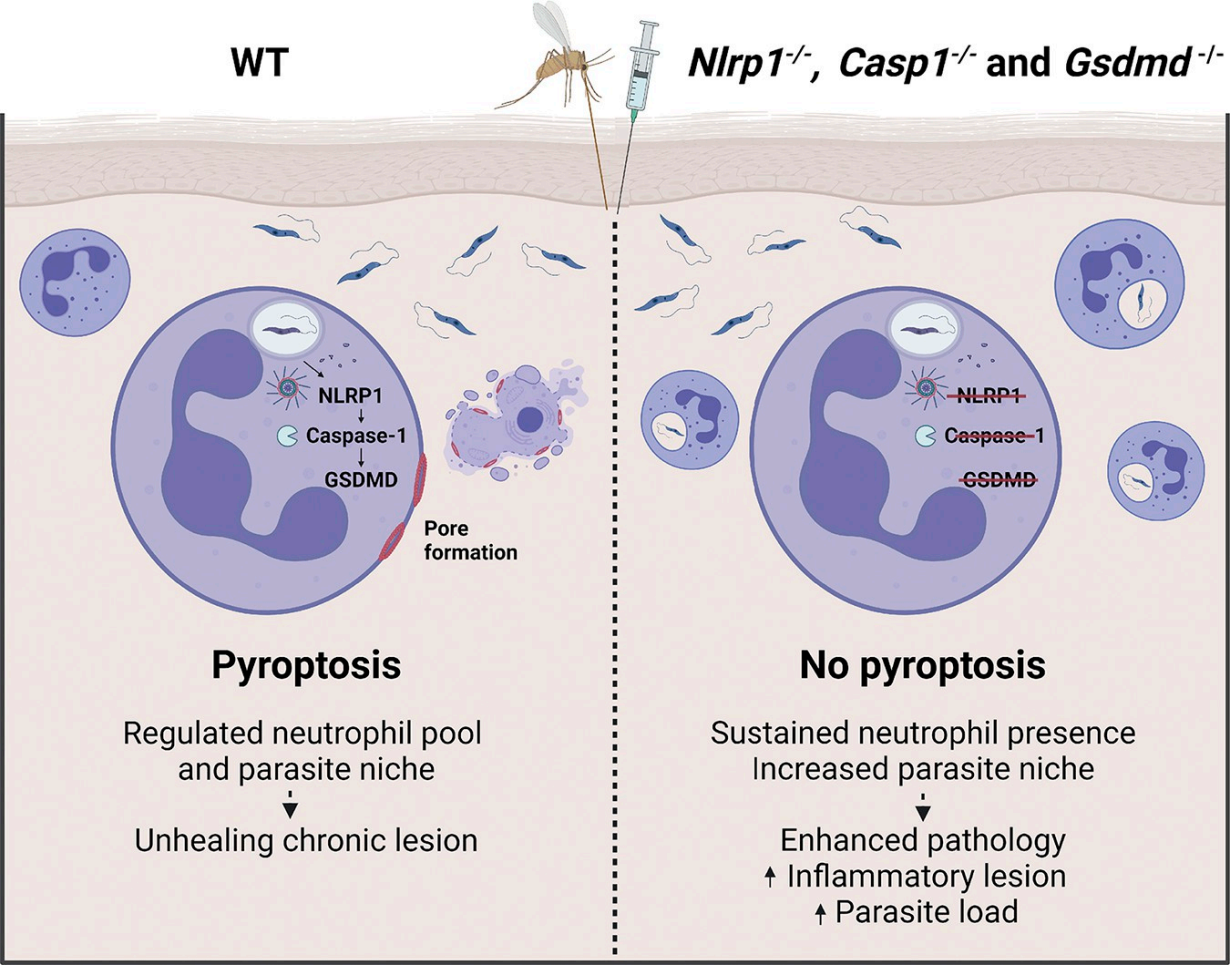
The role of NLRP1-dependent neutrophil pyroptosis during L. mexicana infection. Neutrophils are rapidly recruited upon infection with *L*. *mexicana* and become infected with parasites. Infection leads to activation of the NLRP1 inflammasome and caspase-1 in neutrophils, which cleaves and activates the pore-forming protein GSDMD. GSDMD cleavage leads to neutrophil pyroptosis, which contributes to reduction of the parasite load. GSDMD regulates the early neutrophil parasite niche limiting the subsequent pathology. In absence of GSDMD, more neutrophils provide increased parasite niche and enhance pathology. Figure created with biorender.com.

To further elucidate the role of pyroptosis in the early wave of neutrophils in the long-term pathology, we injected either *Gsdmd*^-/-^ or C57BL/6 neutrophils in *Genista* neutropenic mice at the time of infection and monitored lesion development over 10 weeks (Fig 7H and 7I). *Genista* mice infected with *L*. *mexicana* are able to heal their lesion and clear their parasites, revealing a deleterious role for neutrophils in this infection. Furthermore, healing was linked to the absence of neutrophils during the first days of infection, as a similar healing phenotype was observed following depletion of neutrophils at the onset of infection in C57BL/6 mice [27]. Here, conversely, the transient adoptive transfer of C57BL/6 neutrophils in *Genista* mice early in infection was sufficient to restore the nonhealing phenotype observed in WT C57BL/6 infected mice. Remarkably, mice that received *Gsdmd*^-/-^ neutrophils at the onset of infection developed a significantly larger inflammatory lesion with elevated parasite burden compared to mice that received C57BL/6 neutrophils (Fig 7J and 7K). No difference was observed in CD4^+^IFNγ^+^, CD4^+^IL-4^+^and CD8^+^IFNγ^+^ T cells between *Genista* mice transferred with C57BL/6 or *Gsdmd*^-/-^ neutrophils (S12F Fig). These results show that GSDMD-induced neutrophil pyroptosis plays a crucial role at the onset of infection and underline the significance of neutrophils in shaping the outcome of the disease, as summarized in Fig 8.

## Discussion

Neutrophils are innate immune cells that play key roles in host defense but that can also contribute to pathology. To counteract efficient killing by neutrophils, many pathogens, including *Leishmania* parasites, have developed strategies to escape immune recognition and circumvent destruction, using these cells as Trojan horses to enter silently into the host, favoring pathogen establishment and subsequent dissemination [48,49]. Following infection with most *Leishmania* spp. including *L*. *mexicana*, neutrophils play a deleterious role early in infection [32]. In this study, we explore the role of inflammasome activation and pyroptosis in neutrophils during *L*. *mexicana* infection. We identify the NLRP1, caspase-1, and GSDMD-mediated neutrophil pyroptosis as critical components in the control of the pathology induced by *L*. *mexicana* infection. We also show, using mice with genetic deficiency of GSDMD restricted to neutrophils, that GSDMD-dependent neutrophil pyroptosis plays an important role in the control of lesion exacerbation and the regulation of parasite burden. Adoptive mixed neutrophil transfer experiments further demonstrate that NLRP1-driven neutrophil pyroptosis plays a crucial role during the first days of infection. These data identify GSDMD as a regulator of the early neutrophil niche, reducing parasite dissemination and the subsequent pathology.

Neutrophils were shown to contribute to *L*. *mexicana*-elicited pathology, as genetically neutropenic *Genista* mice or mice transiently depleted of neutrophils at the onset of infection were able to heal their lesion size and clear their parasite burden [27]. Here, we further confirmed the importance of this neutrophil recruitment in disease outcome, showing that conversely, the early transient persistence of neutrophils results in exacerbated disease. It was previously reported that *L*. *mexicana* impairs recruitment of monocytes and monocyte-derived DC, decreasing the effectiveness of the Th1 immune response [27,50,51]. Neutrophils were shown to contribute to the reduced priming of the Th1 response, as neutropenic or mice depleted of neutrophils at the onset of infection better recruited monocytes and monocyte-derived DC, and showed an enhanced protective Th1 immune response where mice are able to heal their lesion [27]. Inversely, the transient early persistence of neutrophils observed here in *L*. *mexicana*-infected *Gsdmd*^-/-^ mice and following the second wave of neutrophil recruitment 21 days p.i. in *Gsdmd*^-/-^ and *Gsdmd*^ΔPMN^ mice, resulted in increased pathogenicity. Of note, we could not detect any difference in T helper response between *Gsdmd*^-/-^, *Gsdmd*^ΔPMN^ and C57BL/6 mice, with similar frequency of IFNγ, IL-4, and IL-17 secreting CD4^+^ T cells observed in dLNs. In line with these data, we did not observe significant changes in macrophage iNOS and arginase levels detected at the site of infection, as analyzed by flow cytometry, suggesting that *L*. *mexicana*-induced pyroptosis does not influence iNOS-derived macrophage killing but rather reduces the neutrophil lifespan and the protective niche they provide for the parasite. Collectively, these data reveal that neutrophils are playing a critical deleterious role contributing to the pathogenicity resulting from *L*. *mexicana* infection.

Upon infection with *Leishmania¸* Toll-like Receptors (TLR) including TLR2, 7/8 and 9 recognize various *Leishmania* pathogen-associated molecular patterns [52–56] and subsequent TLR activation through upregulation of NF-κB can prime the inflammasome. The NLRP3 inflammasome was reported to be activated upon infection with several *Leishmania* spp., with roles that could be either beneficial or detrimental, depending on the infecting *Leishmania* spp. and strain [16]. Most of these studies investigated the activation of the NLRP3 inflammasome in macrophages, the main parasite replicating cells. *L*. *amazonensis* was reported to activate the NLRP3 inflammasome through different pathways including Dectin-1/SYK kinase signaling [57], K^+^ efflux [20], as well as non-canonical activation after lipophosphoglycan (LPG) detection by murine caspase-11 [19]. The NLRP3 inflammasome was also shown to contribute to *L*. *braziliensis* lesion control [58]. On the opposite, following *L*. *major* Seidman infection, the NLRP3 inflammasome was shown to play a detrimental role [17]. In contrast, some *Leishmania* spp. such as *L*. *major* LV39 and *L*. *donovani* failed to show a phenotype upon infection of *Nlrp3*^-/-^ mice [59], but during natural sand fly transmission of *L*. *donovani*, the sand fly gut microbiota was shown to contribute to NLRP3 inflammasome activation [25].

We hypothesized that *L*. *mexicana* could activate the NLRP3 inflammasome with an impact on the pathology. However, infection of *Nlrp3*^*-/-*^ mice showed no phenotypes, with similar lesion development and parasite load compared to control C57BL/6 mice. In this line, infection of *Asc*^-/-^ mice also resulted in similar pathology as infected control mice. The *Leishmania* promastigote stage has a flagellum that could potentially be recognized by the ASC-independent NLRC4 inflammasome, however, infection of *Nlrc4*^-/-^ and control mice resulted in similar pathology. We identified NLRP1 as the inflammasome activated by *L*. *mexicana* infection, in line with the dispensable role of ASC in its activation in mice for this inflammasome [44]. NLRP1 is activated by diverse cell perturbations or molecular entities [60] with a growing list of activators including viral 3C proteases [61–63], ribotoxic stress[64,65], and UV irradiation [66,67] for human NLRP1 and bacterial and protozoan toxins for mouse NLRP1 [22,68–70]. NLRP1 has undergone diversification between humans and mice. In humans, one gene codes for NLRP1 while there are several paralogs for mouse NLRP1 [71]. In addition, hNLRP1 is mostly expressed in epithelial cells and keratinocytes, while its expression has been mostly reported in myeloid cells in mice [44]. NLRP1 transcripts were reported to be upregulated in skin biopsies of human cutaneous leishmaniasis lesions [21]. The mechanisms leading to NLRP1 activation by *L*. *mexicana* remain to be defined. Here, we show that *L*. *mexicana* also induces pyroptosis in human neutrophils. Furthermore, using a NLRP1 agonist, pyroptosis was induced in human neutrophils, a process decreased in presence of caspase-1 inhibition, revealing inflammasome activation. These data suggest that *L*. *mexicana* can induce NLRP1 in neutrophils and other cells in human infected skin, contributing to the modulation of the local inflammatory environment. These findings warrant further investigation. Collectively, the activation of NLRP1-dependent pyroptosis in neutrophils reported here reinforces the importance of this inflammasome in cutaneous inflammatory diseases [72].

Infection of *Gsdmd*^*-/-*^ mice with *L*. *amazonensis* was recently reported to induce lesion exacerbation, a phenotype that depends on NLRP3 inflammasome activation. GSDMD’s protective role was inferred to result from transiently induced potassium efflux in macrophages, due to an atypical GSDMD cleavage product that induced pore formation but no pyroptosis [20]. We infected LPS-primed macrophages with *L*. *mexicana* and detected caspase-1 and GSDMD-dependent IL-1β secretion that was NLRP1-independent. We showed that the NLRP1 inflammasome is protective following *L*. *mexicana* infection and that NLRP1 contributes to neutrophil pyroptosis. In the absence of GSDMD, neutrophils persisted longer at the site of infection during the first days of infection, resulting in a larger number of infected neutrophils. A similar phenotype was observed after adoptive transfer of *Gsdmd*^-/-^, *Caspase1*^-/-^, and *Nlrp1*^-/-^ neutrophils in neutropenic mice. These data establish a role for NLRP1-driven neutrophil pyroptosis early in infection which controls the magnitude of the pathology.

GSDMD has been implicated in NETosis during bacterial infections, where it is activated by caspase-11 or neutrophil elastase [10,45]. However, NET formation can occur independently of GSDMD, depending on the type of NET formation (NETosis *versus* vital NET formation) and the specific stimulus [12,73]. Nuclear decondensation is also observed during caspase-1-dependent neutrophil pyroptosis, but without significant NET expulsion [11]. Consistent with these observations, we observed a small reduction of dsDNA release in *Gsdmd*^*-/-*^ neutrophils, which could be attributed to NETosis or the release of nucleosomes during other types of necrotic cell death, such as pyroptosis. Here, visualizing NETs by microscopy, we show that *L*. *mexicana-*induced NET formation does not depend on caspase-1 or GSDMD. These findings confirm that NET release is independent from caspase-1-driven pyroptosis, as previously observed [11,12].

*L*. *mexicana*-induced neutrophil pyroptosis did not have any impact on other neutrophil functions including ROS production, neutrophil activation and recruitment, showing that activation of GSDMD by *L*. *mexicana* plays a different role in neutrophils. Using adoptive transfer experiments of C57BL/6 and *Gsdmd*^*-/-*^ neutrophils in neutropenic mice, we identified GSDMD as an early regulator of the neutrophil pool upon infection, further stressing the importance of neutrophil pyroptosis in the regulation of *L*. *mexicana* at the onset of infection. Promastigotes rapidly transform into amastigotes predominantly in macrophages, but they can also transform in a subset of neutrophils [46]. Here, we show that amastigotes can induce neutrophil pyroptosis, and that in absence of inflammasome activation, a higher frequency of neutrophils and of parasitized neutrophils is observed, suggesting that the increased persistence of neutrophils may allow *L*. *mexicana* replication in these cells.

*L*. *mexicana*-induced neutrophil pyroptosis could impact parasite survival, via removing the protective niche for parasites and/or pyroptotic microbicidal function. It was reported that exposure to pyroptotic cell lysates damages bacteria and renders them more susceptible to ROS and granule contents [74]. In addition, GSDMD pores attach to acidic phospholipids and cardiolipin in membranes and could have a direct microbicidal effect by forming pores in *L*. *mexicana* membranes as it was shown for bacterial membranes [7,74,75]. Pyroptosis-induced damage decreased infectivity of bacteria to macrophages [74] but not that of *L*. *amazonensis* or *L*. *major* [76,77]. The impact of *L*. *mexicana*-induced pyroptosis in neutrophils and other myeloid cells on parasite infectivity warrants further investigation.

*Leishmania* includes about 20 different spp. that infect humans, inducing cutaneous or visceral diseases. We show that infection of LPS-primed BMNs with a range of different *Leishmania* spp. resulted in distinct GSDMD cleavage in neutrophils. *L*. *mexicana* induced the highest level of GSDMD cleavage and *L*. *panamensis*, another New World spp., also induced GSDMD cleavage. *L*. *major* Seidman-induced pathology was reported to depend on NLRP3 activation and neutrophils [17] and this Old-World spp. also induced GSDMD cleavage in neutrophils, while the other *L*. *major* strains included in this study did not. The other *Leishmania* spp. tested did not induce GSDMD cleavage. Thus, GSDMD cleavage in response to *Leishmania* is not restricted to *L*. *mexicana* and in contrast, some *Leishmania* spp. do not induce neutrophil pyroptosis. The impact of different parasite spp. or strains on GSDMD cleavage in neutrophils may come from distinct parasite features that evolved during evolution. For instance, different levels of virulence factors, such as Leishmaniolysin (Gp63), lipophosphoglycans (LPG), and others, exist between different *Leishmania* spp. and may contribute to observed differences in their activation of inflammasomes. In addition, *Leishmania* strains may have evolved within hosts subjected to anti-parasitic drug therapy, such as *L*. *major* Sd, a strain that was isolated from a multidrug-resistant patient, and that may have developed distinct features compared to other *L*. *major* strains isolated from patients that responded to treatment, and that may involve different innate receptors. Modifications of virulence factors may also occur during extended *in vitro* culture time, and we excluded this for *L*. *mexicana*, as vector competent parasites of the same strain, that successfully developed a mature infection in sand flies, induced similar neutrophil GSDMD cleavage. Furthermore, another *L*. *mexicana* strain isolated from a patient with diffuse cutaneous leishmaniasis [78] induced similar GSDMD cleavage in neutrophils. Thus, GSDMD cleavage in neutrophils differs following infection with distinct *Leishmania* spp. and strains.

Neutrophil GSDMD-dependent pyroptosis is a tightly regulated mechanism, which occurs in response to specific pathogens upon activation of caspase-1 [11,12,79]. Here, we show that caspase-1-dependent neutrophil pyroptosis occurs at later time points than that reported for bacteria. Pyroptosis of mouse and human neutrophils infected with bacteria reaches a maximum within 3–5 hours [11,79], while following infection with *Leishmania*, 10–15 hours are needed for maximum neutrophil pyroptosis, as indicated by the PI uptake kinetics (Fig 4D and 4F) suggesting that *Leishmania* delays the onset of pyroptosis. Macrophages resist *Leishmania*-induced pyroptosis by mechanisms that are unclear [16].

We have focused our study on neutrophils and showed that neutrophil pyroptosis is essential in preventing the exacerbated pathology observed following infection with *L*. *mexicana*. The role of macrophages is also very important in *Leishmania* infection as these cells are the final host cells for the parasites. A small increase in the number of *Leishmania* parasites was previously observed in bone marrow-derived macrophages of *Gsdmd*^-/-^ compared to control C57BL/6 [20], and further investigation of the different mechanisms inducing or preventing pyroptosis in these cells by different *Leishmania* spp. and strains are warranted. During natural infection, the sand fly is transmitting salivary and gut products that also activate inflammasomes [25], thus in addition to NLRP1, other inflammasomes such as NLRP3 may be triggered in neutrophils following infection with *L*. *mexicana* in natural settings.

Collectively, our data shed further light on inflammasome activation and pyroptosis in neutrophils following infection with *L*. *mexicana*, showing a protective role for GSDMD through the regulation of the initial neutrophil niche population. Further understanding of innate immune signaling in neutrophils across *Leishmania* species and how it influences complex host-pathogen interactions could contribute to novel therapeutic approaches to *Leishmania* infections and other neutrophil-driven pathologies.

## Materials and methods

### Ethics statement

Healthy, informed and consenting human blood donors participated in this study. Verbal consent was obtained from the donors. All procedures were approved by the Ethical Committee of the Canton of Vaud (number: CER-VD2017-00182 to FTC) and conducted in accordance with the legislation of the Canton of Vaud, the Swiss Confederation and the Declaration of Helsinki. Animal experimental protocols were approved by the veterinary office regulations of the Canton of Vaud, Switzerland, under authorization number 3476.d to FTC and performed in compliance with Swiss laws for animal protection and the principles of the declaration of Basel.

### Experimental design

In this study, we investigated the effect of NLRP1-induced GSDMD activation in neutrophils during *L*. *mexicana*. Sample size of all assays was based on pilot experiments conducted in our laboratory, to ensure reliable data for statistical analyses and reproducibility. Each experiment was independently replicated at least two to three times, with the number of individual replicates specified in the figure legends. Animals were randomly assigned to different experimental groups.

### Mice

C57BL/6 mice were obtained from Envigo (Cambridgeshire, United Kingdom) or bred in our Epalinges facility. *Nlrp1*^*-/-*^ mice were kindly provided by S. Masters, the Walter and Eliza Hall Institute of Medical Research (Parkville 3052, Australia). These mice are homozygous for the deletion of the entire *Nlrp1* locus (Nlrp1a, Nlrp1b and Nlrp1c) [80]. *Casp1* ^*-/-*^ mice [81] were kindly provided by O. Gross (Institute of Neuropathology, University of Freiburg). *Genista* neutropenic mice were generated by Bernard Malissen [47]. *Nlrp3*^*-/-*^ [82], *Nlrc4*^*-/-*^, *Asc*^*-/-*^ [83], *Gsdmd*^*-/-*^ [5] and *Casp11*^*-/-*^ mice have been described previously. All mouse strains used in this study were either backcrossed to or generated on a C57BL/6 background. All mice were bred and housed in specific pathogen-free facilities at the Epalinges Center. All experiments were performed using 6 to 10-week-old age- and sex-matched mice.

To generate conditional *Gsdmd* knockout (*MRP8*^*cre*^ *Gsdmd*^*lox/lox*^) mice, loxP sites were inserted up and downstream of exon 2 of *Gsdmd* using the 2 following gRNAs (TCCACGGGTTCTATAGACGG’TGG and TCTACTACTCCACTCCTCTG’GGG). Injection of the gRNAs and Cas9 protein into C57BL/6 embryos was done as described before [84]. Biopsies for genotyping were taken at an age of 10–12 days. DNA extraction was performed using the KAPA HotStart Mouse Genotyping Kit according to the manufacturer’s protocol. Genotyping PCR was done using Q5 Polymerase (NEB), carrying out 3 PCR reactions with 2 primer sets: PCR-A (CGCTTCCCTTACCTTGAGCA, GTGTCTAGGTGGTTGTGGGG) covering the binding site of gRNA1, and PCR-B (CAGCCCTACTTGCTCTAGCC, AGCCAAAACACTCCGGTTCT) covering the binding site of gRNA2. The expected fragment sizes were 339 bp for PCR-A and 388 bp for PCR-B in animals harboring a WT allele, and 373 bp and 423 bp in mice harboring the loxP insertions. To obtain neutrophil-specific genetic deletion of GSDMD, GSDMD^lox/lox^ mice were crossed to MRP8^cre/WT^ cre-deleter mice (*MRP8-Cre-ires/GFP*, Jackson AX, stock #021614), deletion of exon 2 was verified by PCR using primers CAGCCCTACTTGCTCTAGCC and GTGTCTAGGTGGTTGTGGGG). Mrp8^cre +^; GSDMD ^flox/WT^ offspring were selected and bred with GSDMD^lox/lox^ mice to generate GSDMD^lox/lox^ MRP8cre ^+/-^ mice. Breeding pairs were established with male MRP8^cre+/-^ and female GSDMD^lox/lox^ mice.

### Parasites

*L*. *mexicana* (WHO strain MYNC/BZ/62/M379) was used in this study. We thank Prof. Scott M. Landfear and Shaden Kamhawi for sharing with us the same strain cultured in their laboratory. *L*. *mexicana DsRed* [85] was kindly donated by Prof. Tony Aebischer (Robert Koch-Institute, Berlin). Both strains were used in all experiments unless indicated. Other used species and strains were *L*. *mexicana* MHOM/ MX/00/Tab3, isolated from a human patient with diffuse leishmaniasis, *L*. *(v) panamensis* MHOM/COL/86/1166LUC, *L*. *amazonensis* (LTB0016), *L*. *guyanensis* (MHOM/BR/75/M4147), *L*. *major LV39*, *L*. *major* Seidman [86] and *L*. *infantum* (MHOM/CH/2016/BELA). *L*. *donovani* (MHOM/SD/62/1S) and *L*. *major* (WR 2885) were donated by Dr. Shaden Kamhawi. Parasites were cultured at 26°C in M199 medium (Gibco), supplemented with 10% fetal calf serum (FCS), 4% HEPES, and 2% PSN (Penicillin, Streptomycin and Neomycin, Bioconcept, Allschwill). Transgenic DsRed parasites were grown under the same conditions, with added 50 μg/ mL Hygromycin B (Sigma-Aldrich). Metacyclic promastigote parasites were selected by density centrifugation, as previously described [87]. Axenic amastigotes were isolated as previously described [46].

### *In vivo* mice infection

10^6^ metacyclic promastigotes were injected intradermally (i.d.) using a 20G needle. In selected cases, a lower dose (10^4^ metacyclic parasites) was injected. Lesion development was monitored biweekly using a caliper, and lesion score was calculated as previously described [36]. In brief, this scoring system considers early inflammation as well as late necrosis, with naïve ears scoring 0, and early inflammation scoring 0.5. Once the lesion outline is clear, lesion size is determined using a caliper. Necrosis in the ear lesion will lead to a maximum score of 8 and immediate sacrifice of the animal.

### Parasite burden determination by limiting dilution assay

To determine the population of living parasites, single-cell suspensions obtained from infected ears were subjected to serial dilution in supplemented M199 media as previously described [37]. Detection of parasites in each well was performed using microscopy and the quantification of parasite number was carried out using the ESTIMFRE software, following established procedures.

### Ear and dLN processing

Ears were collected at the specified time points. The two dermal layers were separated, homogenized, and digested with 0 .2 mg/mL liberase TL (Roche) at 37°C for 2 hours. Digested ears were then filtered through 40 μm filters (Falcon). Draining lymph nodes were recovered and homogenized in DMEM (Gibco). Blood was obtained by cardiac puncture and mixed with 1% EDTA in PBS. Blood, bone marrow, and spleen underwent erythrocyte lysis using ACK buffer.

### Sand fly infection

Four to six days old *Lutzomyia longipalpis* (Jacobina colony) female sand flies reared at the Laboratory of Malaria and Vector Research, NIAID, NIH, were artificially fed as previously described [88]. Briefly, rabbit defibrinated blood (Noble Life Science) was centrifugated at 600*xg* for 10 min. The serum was separated, and complement was inactivated for 30 min at 56°C. Reconstituted blood was spiked with 5x10^6^/ mL *Leishmania mexicana* (MYNC/BZ/62/M379 strain) procyclic promastigotes passaged the day before sand fly infection in Schneider’s media (Gibco) supplemented with 20% fetal bovine serum (Thermo-fisher Scientific) and 1% penicillin/streptomycin (Gibco) and kept at 26°C. Sand flies were starved overnight prior to infection. Post-infection, sand flies were kept on 30% sucrose and maintained at 26°C with 75% humidity with 12 hours light/dark cycle. At day 10–14 post-infection, sand fly midguts (n = 5–10) were dissected in 30 μl of PBS (Lonza), macerated and counted using a hemocytometer. The total number of parasites and percent infectious metacyclic promastigotes per midgut were calculated in two independent experiments.

### Isolation of primary human neutrophils

Whole blood was collected from healthy donors in heparinized tubes at the Interregional Blood Transfusion SRC (Epalinges, Switzerland). Neutrophils were isolated by centrifugation over a polymorphprep (Axis-Shield) gradient as previously described [89]. Purity of neutrophils (>95%) was assessed by cytospin and Diff-quick staining (RAL Diagnostics).

### Flow cytometry

Cell surface antigens in single-cell suspensions were detected with the following rat anti-mouse antibodies: anti-CD45-PerCPCy5.5, anti-CD11c-PeCy7, anti-Ly6C-FITC, anti-Ly6G-PE, and anti-Ly6C-APC (all from BD Bioscience); anti-Ly6G-APC/Cy7 and anti-CD4-AF700 (Biolegend); anti-CD8-APC, anti-CD11b-PB, anti-IFNγ-PeCy7; anti-IFNγ-PE, anti-IL-4-FITC, anti-IL17-PercpCy5.5, anti-Annexin V-PeCy7 (eBioscience), anti-iNOS-AF700 and anti-Arginase1-BV421 (all from eBioscience) diluted in supernatant from 2.4G2 hybridoma cells. Viable cells were gated using Aqua Live/dead (Invitrogen). Parasite infection was determined based on the intrinsic DsRed fluorescence of transgenic parasites. Stained cells were acquired on an LSR-Fortessa (BD Bioscience) and analyzed using FlowJo software (Tree StarA).

### IL-1β ELISA

For IL-1β detection, bone marrow-derived neutrophils and human neutrophils from peripheral blood were seeded at 0.5 x 10^6^ cells per well in a total volume of 200 μL and primed for 4h at 100 ng/mL LPS (mouse) or 1 hour at 500 ng/mL LPS (human) (LPS Ultrapure; Invivogen). After infection with *L*. *mexicana* as described previously, supernatants were collected and released mouse IL-1β (IL-1β ELISA kit, Invitrogen) or human IL-1β (human IL-1β/IL-1F2 ELISA DuoSet Kit, R&D) were analyzed according to the manufacturer’s instructions.

### Neutrophil and macrophage isolation

Femora and tibia of naïve mice were collected, and bone marrow was obtained by centrifugation. Neutrophils were purified by negative magnetic-activated cell sorting (MACS) for dsDNA release assays, to avoid unwanted activation and positive MACS for other experiments where higher purity and lower background apoptosis were preferred. Isolations were performed by using the appropriate anti-Ly6G MicroBeads UltraPure isolation kit, according to the manufacturer’s instructions (Miltenyi Biotec). Neutrophil purity (>95%) was verified flow cytometry. Neutrophils were seeded at 0.5 x 10^6^ cells / well in Optimem + 5% FCS (Gibco). For macrophage differentiation, bone marrow cells were cultured for 7 days in RPMI (10% heat-inactivated fetal bovine serum, 1% HEPES (Amimed) and 100U/ mL penicillin and 100μg/ mL streptomycin, Invitrogen) with 25ng/ mL M-CSF (Immunotools- 12343117) to generate BM-derived macrophages. LPS priming (LPS Ultrapure; Invivogen) was performed at 100 ng/ mL in OptiMEM (Gibco) + 5% FCS for 4h at 37°C when indicated.

### Cell Death and permeabilization measurements

Cell-membrane permeabilization was determined by measuring the uptake of Propidium Iodide (PI) (1 μg/mL; Thermo Fisher Scientific) over time using a fluorescent plate reader (Cytation5; Biotek) or an IncuCyte live-cell analysis system (Sartorius). Cell lysis was quantified by measuring LDH release into the supernatant (Cytotx kit, Promega). Both values of PI and LDH were calculated as a percentage of a 100% lysis control and normalized to the respective untreated sample.

### Immunoblotting

Cells were lysed using lysis buffer containing protease inhibitor cocktail (Complete Ultra Tablets, Roche). Protein separation was performed using 12% gels and transferred onto nitrocellulose conform standard procedures. Antibodies for immunoblots were against GSDMD (EPR19828; Abcam; 1:1000) and beta-actin (Sigma; 1:5000).

### DsDNA release measurements

Double-stranded DNA release was quantified in the supernatants by Picogreen detection as previously described [56]. Briefly, neutrophils were obtained through negative MACS selection and primed with GM-CSF for 20 min at 37°C, prior to infection with stationary-phase *L*. *mexicana* promastigotes. After 4 hours of infection, stimulation was stopped with the addition of DNAse I (20 μg/mL; Roche) and EDTA (2.5 mM). Supernatants were collected, transferred to 96-well plates (Perkin Elmer), and light-sensitive Picogreen dye was added. DsDNA content was then measured at 480nm excitation and 520 nm emission using a spectrophotometer (Molecular Devices, SpectraMax MiniMax 300).

### ROS detection

Total reactive oxygen species (ROS) production by bone marrow-derived neutrophils was measured through a luminol-based chemiluminescence assay as previously described [56]. Briefly, BMNs were incubated in X-vivo (Lonza) medium in a white opaque 96-well plate (White Opaque 96-well Microplate, PerkinElmer) and infected with *L*. *mexicana* promastigotes. ROS production was measured by adding 20 μg/mL of luminol (Carbosynth) and measuring chemiluminescence at all wavelengths over time, using a Spectramax plate reader (Molecular Devices, SpectraMax MiniMax 300). To measure ROS by flow cytometry, BMNs were incubated with 1.2 μM DHR123 molecular probe (Thermo Fisher Scientific) for 45 min at 37°C.

### Neutrophil adoptive transfer

BMNs from the indicated genotypes were stained with either CMRA (Cell tracker Orange, Life Technologies) or CMFDA (Cell tracker Green, Life Technologies), for 15 min at 37°C, in DMEM (Gibco) containing 1% FBS. For each replicate, dyes were switched to ensure no bias due to dye color. After staining, neutrophils were counted and mixed at an equal 1:1 ratio. At the time of infection, 10^6^ neutrophils were injected with 10^6^ metacyclic promastigotes into the ear dermis of *Genista* mice. Following infection in WT mice, neutrophils peak at the site of infection between 4 and 24 hours [27,89]. 24 hours after neutrophil adoptive transfer and co-infection, at a time that may correspond to 28 hours or more p.i. at the site of infection in WT C57BL/6 mice, mice were sacrificed, and ears and blood were processed as previously described.

### Immunofluorescence staining and confocal microscopy

OCT cryosections of infected ears were prepared and thawed for 30 minutes at room temperature before fixation for 10 minutes in 4% PFA. After 30 minutes in blocking buffer (0.5% BSA, 5% donkey serum, 0.3% Triton X-100, 0,1% NaN3), slides were incubated with primary Ly6G^+^ antibody (Biolegend) overnight at 4°C. Washing was performed for 30 minutes with 0.3% Triton X-100 in PBS. Secondary antibodies were diluted 1:500 in blocking buffer and staining was performed for 1 hour at RT. After washing, slides were mounted using Fluoromount-G, containing DAPI (Invitrogen). Images were acquired with an inverted confocal microscope Zeiss LSM 880 with Airyscan and processed in Imaris 9.0 (Bitplane). Total area of DAPI, Ly6G^+^ and parasite dsRed was quantified, and total area of Ly6G^+^ and parasite dsRed was normalized to DAPI in each field of view.

### Statistical analysis

Statistical analyses were performed using Prism 8 (GraphPad) software. When data sets were normally distributed, parametric unpaired t-tests were used, if not, a non-parametric Mann-Whitney U test was used to analyze the data. Adoptive transfer of neutrophils of several genotypes was analyzed with Wilcoxon matched pairs signed rank test. When more than 2 groups were considered, a parametric 1-way ANOVA or non-parametric Kruskal-Wallis test was performed, comparing each group back to the control variable. Long-term mice infections, as well as kinetic PI absorbance, were analyzed using a 2-way ANOVA model with a time variable. P-values were considered significant at p ≤ 0.05.

## Supporting information

S1 Fig The NLRP3 inflammasome does not influence the *L*. *mexicana* induced pathology. *Nlrp3*^*-/-*^ and C57BL/6 mice were infected i.d with 10^6^ *L*. *mexicana* promastigotes and lesion development was measured over time. **(A)** General flow cytometry gating strategy for infected ears. **(B)** Corresponding frequencies of ear CD45^**+**^ cells, CD45^+^CD11b^+^Ly6G^+^ neutrophils, CD45^+^CD11b^+^Ly6C^+^ monocytes, and CD45^+^CD11C^+^ dendritic cells. Data are shown as mean ± SD and statistical differences were analyzed with a Mann-Whitney U test. Data are representative of ≥3 experiments with n≥4/group. ***p <0.001
(TIF)

S2 Fig Non-canonical inflammasome activation and NLRC4 do not influence *L*. *mexicana* induced pathology. **(A)** *Casp11*^-/-^ and C57BL/6 mice were infected with *L*. *mexicana* promastigotes and lesion score was measured over time. **(B)** Lesion size at 9 weeks p.i. and **(C)** parasite load at the site of infection as determined by LDA. **(D)** The number and frequency of CD45^**+**^ cells were analyzed by flow cytometry. **(E)** The number of CD45^+^CD11b^+^Ly6G^+^ neutrophils, CD45^+^CD11b^+^Ly6C^+^ monocytes, and CD45^+^CD11C^+^ dendritic cells, as determined by flow cytometry. **(F)** *Asc*^-/-^and C57BL/6 control mice were infected with *L*. *mexicana* promastigotes and lesion score was measured over time. **(G)** Lesion size and **(H)** parasite burden at 9 weeks p.i. **(I)** The number and frequency of CD45^**+**^ cells and **(J)** the number of CD45^+^CD11b^+^Ly6G^+^ neutrophils, CD45^+^CD11b^+^Ly6C^+^ monocytes, and CD45^+^CD11C^+^ dendritic cells at the site of infection. **(K)** *Nlrc4*^-/-^ and C57BL/6 control mice were similarly infected with *L*. *mexicana* and lesion score was measured over the course of infection. **(L)** Fifteen weeks p.i., the lesion size was measured, with parasite burden **(M)**, determined by LDA, and **(N)** the number and frequency of dermal CD45^**+**^ cells were determined by flow cytometry. **(O)** The numbers of CD45^+^CD11b^+^Ly6G^+^ neutrophils, CD45^+^CD11b^+^Ly6C^+^ monocytes, and CD45^+^CD11C^+^ dendritic cells were analyzed by flow cytometry. Data are shown as mean ± SD and statistical differences in lesion development were analyzed with a 2-way ANOVA. Data are representative of ≥3 experiments with n≥4/group.
(TIF)

S3 Fig The NLRP1 inflammasome does not influence the adaptive immune response during *L mexicana* infection. **(A)** *Nlrp1*^*-/-*^ and C57BL/6 control mice were infected i.d. with metacyclic *L*. *mexicana* promastigotes and 8 weeks p.i., and the frequency of CD45^+^CD11b^+^Ly6G^+^ neutrophils, CD45^+^CD11b^+^Ly6C^+^ monocytes, and CD45^+^CD11c^+^ dendritic cells in infected ears was analyzed by flow cytometry. **(B)** The frequency of CD4^+^ IFN-γ^+^, CD4^+^ IL-4^+^, and CD8^+^ IFN-γ^+^ T cells in draining lymph nodes (dLN) and **(C)** ears was analyzed by flow cytometry.
(TIF)

S4 Fig Caspase-1 and GSDMD do not influence the adaptive immune response during *L mexicana* infection. **(A)** *Casp1*^*-/-*^ and C57BL/6 control mice were infected i.d. with metacyclic *L*. *mexicana* promastigotes and 7 weeks p.i. the frequency of CD45^+^CD11b^+^Ly6G^+^ neutrophils, CD45^+^CD11b^+^Ly6C^+^ monocytes, and CD45^+^CD11c^+^ dendritic cells in infected ears was analyzed by flow cytometry. **(B)** The frequency of CD4^+^ IFN-γ^+^, CD4^+^ IL-4^+^, and CD8^+^ IFN-γ^+^ T cells present in dLN and **(C)** infected ears was analyzed by flow cytometry**. (D)** *Gsdmd*^*-/-*^ and C57BL/6 control mice were similarly infected for 8 weeks and the frequency of CD45^+^CD11b^+^Ly6G^+^ neutrophils, CD45^+^CD11b^+^Ly6C^+^ monocytes, and CD45^+^CD11c^+^ dendritic cells in infected ears was determined by flow cytometry. **(E)** The frequency of CD4^+^ IFN-γ^+^, CD4^+^ IL-4^+^, and CD8^+^ IFN-γ^+^ T cells in dLN and **(F)** ear was similarly analyzed by flow cytometry. Data are shown as mean ± SD and statistical differences between groups were analyzed by Mann-Whitney U-test and are representative of ≥3 experiments with n≥4/group. *p <0.05.
(TIF)

S5 Fig Gasdermin D reduces *L*. *mexicana-*induced pathology and parasite burden following infection with a low dose of parasites. **(A)** *Gsdmd*^*-/-*^and C57BL/6 were infected i.d. with a low dose (10^4^) of *L*. *mexicana* and lesion progression was assessed over 16 weeks. **(B)** Lesion size and parasite burden at 16 weeks p.i., at the infection site (**C)** and in the dLN **(D)**. Data are shown as mean ± SEM and are representative of ≥2 experiments, n≥4/group. Statistical differences in lesion development were analyzed with a 2-way ANOVA with repeated measures, and parasite number and cell percentages using a Mann-Whitney U-test. *p <0.05; **p <0.01
(TIF)

S6 Fig Macrophage production of IL-1β upon *L*. *mexicana* infection is caspase-1 and GSDMD-dependent. **(A)** LPS-primed, C57BL/6, *Nlrp1*^*-/-*^, *Casp1*^*-/-*^, *Casp11*^*-/*^ and *Gsdmd*^*-/-*^ bone marrow-derived macrophages (BMDMs) were infected with *L*. *mexicana* promastigotes at an MOI of 2, 5 and 10 for 16h, or exposed to nigericin for 4 hours, as a positive control. IL-1β concentration was measured in cell supernatants by ELISA. Data are shown as mean ± SD and are representative of n>3 experiments. 2-way ANOVA with Dunnett’s multiple comparison, *p <0.05; **p <0.01, ***p>0001.
(TIF)

S7 Fig *L*. *mexicana* develops mature infections in *Lutzomyia longipalpis* sand flies. **(A**) To assess vector competency of *Leishmania mexicana* in *Lutzomyia longipalpis*, sand flies were infected with 5x10^6^/ mL procyclic promastigote parasites. Infection was assessed by dissecting 5–10 sand fly midguts at day 10 post-infection. The total number of parasites and the percentage of infectious metacyclic promastigotes per midgut were counted. Data are representative of two independent experiments and are represented as mean ± SD. (**B**) LPS-primed C57BL/6 BMNs were infected with vector-competent *L*. *mexicana* metacyclic promastigotes or the same strain maintained in standard growing media and passaged in BALB/c mice. BMNs were infected for 16 hours at MOI 5 or 10 and cell lysates were subjected to immunoblotting for GSDMD cleavage and β-actin. Nigericin was used as a positive control. Data are representative for n≥2 independent experiments.
(TIF)

S8 Fig *L*. *mexicana* infection does not impact non-lytic apoptosis. (**A)** LPS-primed BMNs of C57BL/6 and *Gsdmd*^*-/-*^ were infected at an MOI of 2, 5, and 10, and 16 hours later, neutrophils were stained with Annexin-V and DAPI to assess apoptosis by flow cytometry. A representative plot is shown and **(B)** the relative frequency of viable, early apoptotic, late apoptotic, and necrotic cells is given. Data are shown as mean ± SD and are representative of ≥2 experiments.
(TIF)

S9 Fig NET formation upon *L*. *mexicana* infection is caspase-1 and GSDMD-independent. **(A)** BMNs were isolated and infected with *L*. *mexicana* at an MOI5. Four hours later, dsDNA release was analyzed by Picogreen assay. PMA was used as a positive control and BMNs treated with DNAse I as a negative control. Results are shown as a pool of 3 replicates. n = 3/group. **(B)** *Ex vivo* NET formation of BMNs exposed for 4 hours to medium (uninfected), *L*. *mexicana* (MOI5) and PMA. Samples were fixed, stained for MPO and DNA (DAPI) and analyzed by confocal microscopy. Representative confocal microscopy images are shown. On the right, the frequency of netting neutrophils is shown, with NETs defined by colocalization of decondensed DNA and MPO. n = 9 random fields/group. **(C)** *Ex vivo* NET formation of isolated human neutrophils exposed for 4 hours to medium, *L*. *mexicana* (MOI5), or PMA. Representative pictures and frequency of netting neutrophils is shown. n = 9 random fields/group. Scale bar: 20 μm. Data are shown as mean ± SD. One representative experiment out of two is shown in each graph **(B, C)**. *p <0.05, **p <0.01, ***p <0.001, ns: non-significant, as determined by Kruskal-Wallis (B) and Mann-Whitney U-test (A, C).
(TIFF)

S10 Fig Impact of GSDMD on neutrophil function. **(A)** BMNs of *Gsdmd*^*-/-*^ and C57BL/6 mice were infected with *L*. *mexicana* promastigotes at MOI 5 and reactive oxygen species (ROS) production was measured over 2h by the addition of luminol. Values are represented as mean relative light units (RLU) ± SD over time. PMA was used as a positive control, with non-stimulated (NS) as a negative control. **(B)** Area under the curve (AUC) ± SD of representative curves. Data are shown as mean ± SD and representative of ≥3 experiments. **(C)** LPS-primed *Gsdmd*^*-/-*^ and C57BL/6 BMNs were infected with dsRed^+^-expressing *L*. *mexicana* promastigotes at the indicated MOI and the frequency of *L*. *mexicana*-dsRed^+^ neutrophils was analyzed by flow cytometry, at 2- and 24-hours p.i. respectively. **(D)** Neutrophil activation status and degranulation was analyzed by flow cytometry, showing the mean fluorescence intensity (MFI) of CD11b, CD62L, and CD63. Ionomycin was used as a positive control. Data are represented as mean ± SD and are representative of ≥2 experiments. NS (non-stimulated). 2-way ANOVA with Dunnett’s multiple comparison *p <0.05; **p <0.01.
(TIF)

S11 Fig GSDMD regulates the neutrophil niche during the first days and weeks of infection. **(A)** C57BL/6 and *Gsdmd*^*-/-*^ mice were infected i.d with metacyclic dsRed^+^ *L*. *mexicana* promastigotes and frequency of CD45^+^CD11b^+^Ly6G^+^ neutrophils was determined by flow cytometry at the indicated timepoints. **(B)** Frequency of infected CD45^+^CD11b^+^Ly6G^+^dsRed^+^ neutrophils at 24, 48, 72 hours and 21 days p.i. Data is representative of n≥2 independent experiments and differences were analyzed by Mann-Whitney U-test. *p <0.05.
(TIF)

S12 Fig Specific depletion of GSDMD in neutrophils has no impact on adaptive immune response. **(A)** Left panel: representative immunohistology pictures of PBS-injected ear stained for Ly6G^+^ neutrophils (green), and DAPI (blue), serving as technical negative control for DsRed. Right panel: technical negative control for anti-Ly6G antibody, showing the absence of Ly6G^+^ neutrophils (green) upon omission of primary antibody, DAPI (grey), and dsRed^+^ parasites. Scale bar, 50 μm. **(B)** *Gsdmd*^*-/-*^, *Gsdmd*^ΔPMN^, and WT, mice were infected i.d. with *L*. *mexicana* promastigotes. The frequency of CD45^+^ cells, CD45^+^CD11b^+^Ly6G^+^ neutrophils, CD45^+^CD11b^+^Ly6C^+^ monocytes, and CD45^+^CD11c^+^ dendritic cells at 11 weeks p.i. was analyzed by flow cytometry. **(C)** The frequency of CD4^+^ IFN-γ^+^, CD4^+^ IL-4^+^, CD4^+^ IL-17^+^ and CD8^+^ IFN-γ^+^ T cells in draining lymph nodes (dLN) was analyzed by flow cytometry at 11 weeks p.i., and **(D)** Mice were similarly infected and 21 days later, the frequency of CD4^+^ IFN-γ^+^ and CD4^+^ IL-4^+^ T cells was analyzed by flow cytometry **(E)** The frequency of arginase (Arg^+^) and iNOS positive CD45^+^CD11b^+^F480^+^ macrophages, as determined by flow cytometry. **(F)** C57BL/6 and *Gsdmd*^-/-^ BMNs were isolated and adoptively transferred at the time of infection in two separate groups of *Genista* mice. The frequency of CD4^+^ IFN-γ^+^, CD4^+^ IL-4^+^ and CD8^+^ IFN-γ^+^ T cells after 10 weeks p.i. Data are representative of n≥2 independent experiments. Differences in cell populations were analyzed with Kruskall-Wallis with Dunn’s multiple comparisons test **(B, C, D, E)** or Mann-Whitney U test **(D)**. **p <0.01.
(TIF)

S1 Data Supporting file: The raw data supporting each of the manuscript Figures are included in this Figure. (XLSX)

## References

[1] Martinon F, Burns K, Tschopp J. The inflammasome: a molecular platform triggering activation of inflammatory caspases and processing of proIL-beta. Mol Cell. 2002;10(2):417–26. doi: 10.1016/s1097-2765(02)00599-3 12191486

[2] Broz P, Dixit VM. Inflammasomes: mechanism of assembly, regulation and signalling. Nature Reviews Immunology. 2016;16(7):407–20. doi: 10.1038/nri.2016.58 27291964

[3] He WT, Wan H, Hu L, Chen P, Wang X, Huang Z, et al. Gasdermin D is an executor of pyroptosis and required for interleukin-1β secretion. Cell Res. 2015;25(12):1285–98. 26611636 10.1038/cr.2015.139 PMC4670995

[4] Shi J, Zhao Y, Wang K, Shi X, Wang Y, Huang H, et al. Cleavage of GSDMD by inflammatory caspases determines pyroptotic cell death. Nature. 2015;526(7575):660–5. doi: 10.1038/nature15514 26375003

[5] Kayagaki N, Stowe IB, Lee BL, O’Rourke K, Anderson K, Warming S, et al. Caspase-11 cleaves gasdermin D for non-canonical inflammasome signalling. Nature. 2015;526(7575):666–71. doi: 10.1038/nature15541 26375259

[6] Rühl S, Broz P. Caspase-11 activates a canonical NLRP3 inflammasome by promoting K(+) efflux. Eur J Immunol. 2015;45(10):2927–36. doi: 10.1002/eji.201545772 26173909

[7] Liu X, Zhang Z, Ruan J, Pan Y, Magupalli VG, Wu H, et al. Inflammasome-activated gasdermin D causes pyroptosis by forming membrane pores. Nature. 2016;535(7610):153–8. doi: 10.1038/nature18629 27383986 PMC5539988

[8] Bergsbaken T, Fink SL, Cookson BT. Pyroptosis: host cell death and inflammation. Nat Rev Microbiol. 2009;7(2):99–109. doi: 10.1038/nrmicro2070 19148178 PMC2910423

[9] Karmakar M, Minns M, Greenberg EN, Diaz-Aponte J, Pestonjamasp K, Johnson JL, et al. N-GSDMD trafficking to neutrophil organelles facilitates IL-1β release independently of plasma membrane pores and pyroptosis. Nat Commun. 2020;11(1):2212. 32371889 10.1038/s41467-020-16043-9 PMC7200749

[10] Chen KW, Monteleone M, Boucher D, Sollberger G, Ramnath D, Condon ND, et al. Noncanonical inflammasome signaling elicits gasdermin D–dependent neutrophil extracellular traps. Science Immunology. 2018;3(26):eaar6676-eaar. doi: 10.1126/sciimmunol.aar6676 30143554

[11] Santoni K, Pericat D, Gorse L, Buyck J, Pinilla M, Prouvensier L, et al. Caspase-1-driven neutrophil pyroptosis and its role in host susceptibility to Pseudomonas aeruginosa. PLoS Pathog. 2022;18(7):e1010305. doi: 10.1371/journal.ppat.1010305 35849616 PMC9345480

[12] Chauhan D, Demon D, Vande Walle L, Paerewijck O, Zecchin A, Bosseler L, et al. GSDMD drives canonical inflammasome-induced neutrophil pyroptosis and is dispensable for NETosis. EMBO reports. 2022;23(10):e54277. doi: 10.15252/embr.202154277 35899491 PMC9535806

[13] Dubyak GR, Miller BA, Pearlman E. Pyroptosis in neutrophils: Multimodal integration of inflammasome and regulated cell death signaling pathways. Immunological Reviews. 2023;314(1):229–49. doi: 10.1111/imr.13186 36656082 PMC10407921

[14] Burza S, Croft SL, Boelaert M. Leishmaniasis. The Lancet. 2018;392(10151):951–70. 10.1016/S0140-6736(18)31204-2 30126638

[15] Faria MS, Reis FC, Lima AP. Toll-like receptors in leishmania infections: guardians or promoters? J Parasitol Res. 2012;2012:930257. doi: 10.1155/2012/930257 22523644 PMC3317170

[16] Zamboni DS, Sacks DL. Inflammasomes and Leishmania: in good times or bad, in sickness or in health. Current Opinion in Microbiology. 2019;52:70–6. doi: 10.1016/j.mib.2019.05.005 31229882 PMC6910972

[17] Charmoy M, Hurrell BP, Romano A, Lee SH, Ribeiro-Gomes F, Riteau N, et al. The Nlrp3 inflammasome, IL-1beta, and neutrophil recruitment are required for susceptibility to a nonhealing strain of Leishmania major in C57BL/6 mice. Eur J Immunol. 2016;46(4):897–911. 26689285 10.1002/eji.201546015 PMC4828310

[18] Chaves MM, Sinflorio DA, Thorstenberg ML, Martins MDA, Moreira-Souza ACA, Rangel TP, et al. Non-canonical NLRP3 inflammasome activation and IL-1β signaling are necessary to L. amazonensis control mediated by P2X7 receptor and leukotriene B4. PLoS Pathog. 2019;15(6):e1007887. 31233552 10.1371/journal.ppat.1007887 PMC6622556

[19] de Carvalho RVH, Andrade WA, Lima-Junior DS, Dilucca M, de Oliveira CV, Wang K, et al. Leishmania Lipophosphoglycan Triggers Caspase-11 and the Non-canonical Activation of the NLRP3 Inflammasome. Cell Reports. 2019;26(2):429–37.e5. 30625325 10.1016/j.celrep.2018.12.047 PMC8022207

[20] de Sá KSG, Amaral LA, Rodrigues TS, Ishimoto AY, de Andrade WAC, de Almeida L, et al. Gasdermin-D activation promotes NLRP3 activation and host resistance to Leishmania infection. Nat Commun. 2023;14(1):1049. doi: 10.1038/s41467-023-36626-6 36828815 PMC9958042

[21] Gupta G, Santana AKM, Gomes CM, Turatti A, Milanezi CM, Bueno Filho R, et al. Inflammasome gene expression is associated with immunopathology in human localized cutaneous leishmaniasis. Cellular Immunology. 2019;341:103920. doi: 10.1016/j.cellimm.2019.04.008 31078283

[22] Gorfu G, Cirelli KM, Melo MB, Mayer-Barber K, Crown D, Koller BH, et al. Dual Role for Inflammasome Sensors NLRP1 and NLRP3 in Murine Resistance to Toxoplasma gondii. mBio. 2014;5(1): doi: 10.1128/mBio.01117-13 24549849 PMC3944820

[23] Tacchini-Cottier F, Zweifel C, Belkaid Y, Mukankundiye C, Vasei M, Launois P, et al. An Immunomodulatory Function for Neutrophils During the Induction of a CD4+ Th2 Response in BALB/c Mice Infected with Leishmania major1. The Journal of Immunology. 2000;165(5):2628–36. 10946291 10.4049/jimmunol.165.5.2628

[24] Peters NC, Egen JG, Secundino N, Debrabant A, Kimblin N, Kamhawi S, et al. In vivo imaging reveals an essential role for neutrophils in leishmaniasis transmitted by sand flies. Science. 2008;321(5891):970–4. doi: 10.1126/science.1159194 18703742 PMC2606057

[25] Dey R, Joshi AB, Oliveira F, Pereira L, Guimarães-Costa AB, Serafim TD, et al. Gut Microbes Egested during Bites of Infected Sand Flies Augment Severity of Leishmaniasis via Inflammasome-Derived IL-1β. Cell Host & Microbe. 2018;23(1):134–43.e6. 29290574 10.1016/j.chom.2017.12.002 PMC5832060

[26] Serafim TD, Coutinho-Abreu IV, Dey R, Kissinger R, Valenzuela JG, Oliveira F, et al. Leishmaniasis: the act of transmission. Trends in Parasitology. 2021;37(11):976–87. doi: 10.1016/j.pt.2021.07.003 34389215

[27] Hurrell BP, Schuster S, Grün E, Coutaz M, Williams RA, Held W, et al. Rapid Sequestration of Leishmania mexicana by Neutrophils Contributes to the Development of Chronic Lesion. PLOS Pathogens. 2015;11(5):e1004929. doi: 10.1371/journal.ppat.1004929 26020515 PMC4447405

[28] Hickey MJ, Kubes P. Intravascular immunity: the host-pathogen encounter in blood vessels. Nat Rev Immunol. 2009;9(5):364–75. doi: 10.1038/nri2532 19390567

[29] Gabriel C, McMaster WR, Girard D, Descoteaux A. Leishmania donovani promastigotes evade the antimicrobial activity of neutrophil extracellular traps. J Immunol. 2010;185(7):4319–27. doi: 10.4049/jimmunol.1000893 20826753

[30] Guimarães-Costa AB, Nascimento MTC, Froment GS, Soares RPP, Morgado FN, Conceição-Silva F, et al. Leishmania amazonensis promastigotes induce and are killed by neutrophil extracellular traps. Proceedings of the National Academy of Sciences. 2009;106(16):6748–53. doi: 10.1073/pnas.0900226106 19346483 PMC2672475

[31] Regli IB, Passelli K, Hurrell BP, Tacchini-Cottier F. Survival Mechanisms Used by Some Leishmania Species to Escape Neutrophil Killing. Front Immunol. 2017;8:1558. doi: 10.3389/fimmu.2017.01558 29250059 PMC5715327

[32] Passelli K, Billion O, Tacchini-Cottier F. The Impact of Neutrophil Recruitment to the Skin on the Pathology Induced by Leishmania Infection. Frontiers in Immunology. 2021;12. 10.3389/fimmu.2021.649348 PMC7957080 33732265

[33] van Zandbergen G, Klinger M, Mueller A, Dannenberg S, Gebert A, Solbach W, et al. Cutting edge: neutrophil granulocyte serves as a vector for Leishmania entry into macrophages. J Immunol. 2004;173(11):6521–5. doi: 10.4049/jimmunol.173.11.6521 15557140

[34] Chaves MM, Lee SH, Kamenyeva O, Ghosh K, Peters NC, Sacks D. The role of dermis resident macrophages and their interaction with neutrophils in the early establishment of Leishmania major infection transmitted by sand fly bite. PLOS Pathogens. 2020;16(11):e1008674. doi: 10.1371/journal.ppat.1008674 33137149 PMC7660907

[35] Harrington V, Gurung P. Reconciling protective and pathogenic roles of the NLRP3 inflammasome in leishmaniasis. Immunol Rev. 2020;297(1):53–66. doi: 10.1111/imr.12886 32564424 PMC7643606

[36] Schuster S, Hartley M-A, Tacchini-Cottier F, Ronet C. A scoring method to standardize lesion monitoring following intra-dermal infection of Leishmania parasites in the murine ear. Frontiers in cellular and infection microbiology. 2014;4:67–. doi: 10.3389/fcimb.2014.00067 24904841 PMC4035563

[37] Passelli K, Prat-Luri B, Merlot M, Goris M, Mazzone M, Tacchini-Cottier F. The c-MET receptor tyrosine kinase contributes to neutrophil-driven pathology in cutaneous leishmaniasis. PLoS Pathog. 2022;18(1):e1010247. doi: 10.1371/journal.ppat.1010247 35041723 PMC8797216

[38] Zhang P, Liu Y, Hu L, Huang K, Hong M, Wang Y, et al. NLRC4 inflammasome–dependent cell death occurs by a complementary series of three death pathways and determines lethality in mice. Science Advances. 2021;7(43):eabi9471. doi: 10.1126/sciadv.abi9471 34678072 PMC8535822

[39] Van Opdenbosch N, Gurung P, Vande Walle L, Fossoul A, Kanneganti T-D, Lamkanfi M. Activation of the NLRP1b inflammasome independently of ASC-mediated caspase-1 autoproteolysis and speck formation. Nature Communications. 2014;5(1):3209. doi: 10.1038/ncomms4209 24492532 PMC3926011

[40] Broz P, von Moltke J, Jones JW, Vance RE, Monack DM. Differential Requirement for Caspase-1 Autoproteolysis in Pathogen-Induced Cell Death and Cytokine Processing. Cell Host & Microbe. 2010;8(6):471–83. doi: 10.1016/j.chom.2010.11.007 21147462 PMC3016200

[41] Robert Hollingsworth L, David L, Li Y, Griswold AR, Ruan J, Sharif H, et al. Mechanism of filament formation in UPA-promoted CARD8 and NLRP1 inflammasomes. Nature Communications. 2021;12(1):189. doi: 10.1038/s41467-020-20320-y 33420033 PMC7794386

[42] Kimblin N, Peters N, Debrabant A, Secundino N, Egen J, Lawyer P, et al. Quantification of the infectious dose of Leishmania major transmitted to the skin by single sand flies. Proc Natl Acad Sci U S A. 2008;105(29):10125–30. doi: 10.1073/pnas.0802331105 18626016 PMC2481378

[43] Galindo-Sevilla N, Soto N, Mancilla J, Cerbulo A, Zambrano E, Chavira R, et al. Low serum levels of dehydroepiandrosterone and cortisol in human diffuse cutaneous leishmaniasis by Leishmania mexicana. Am J Trop Med Hyg. 2007;76(3):566–72. 17360885

[44] Bauernfried S, Hornung V. Human NLRP1: From the shadows to center stage. J Exp Med. 2022;219(1). doi: 10.1084/jem.20211405 34910085 PMC8679799

[45] Sollberger G, Choidas A, Burn GL, Habenberger P, Di Lucrezia R, Kordes S, et al. Gasdermin D plays a vital role in the generation of neutrophil extracellular traps. Science immunology. 2018;3(26):eaar6689-eaar. 10.1126/sciimmunol.aar6689 30143555

[46] Hurrell BP, Beaumann M, Heyde S, Regli IB, Muller AJ, Tacchini-Cottier F. Frontline Science: Leishmania mexicana amastigotes can replicate within neutrophils. J Leukoc Biol. 2017;102(5):1187–98. doi: 10.1189/jlb.4HI0417-158R 28798144

[47] Ordoñez-Rueda D, Jönsson F, Mancardi DA, Zhao W, Malzac A, Liang Y, et al. A hypomorphic mutation in the Gfi1 transcriptional repressor results in a novel form of neutropenia. Eur J Immunol. 2012;42(9):2395–408. doi: 10.1002/eji.201242589 22684987

[48] Laskay T, van Zandbergen G, Solbach W. Neutrophil granulocytes—Trojan horses for Leishmania major and other intracellular microbes? Trends Microbiol. 2003;11(5):210–4. doi: 10.1016/s0966-842x(03)00075-1 12781523

[49] Kobayashi SD, Malachowa N, DeLeo FR. Neutrophils and Bacterial Immune Evasion. J Innate Immun. 2018;10(5–6):432–41. doi: 10.1159/000487756 29642066 PMC6784029

[50] Petritus PM, Manzoni-de-Almeida D, Gimblet C, Gonzalez Lombana C, Scott P. Leishmania mexicana Induces Limited Recruitment and Activation of Monocytes and Monocyte-Derived Dendritic Cells Early during Infection. PLOS Neglected Tropical Diseases. 2012;6(10):e1858. doi: 10.1371/journal.pntd.0001858 23094119 PMC3475671

[51] Contreras I, Estrada JA, Guak H, Martel C, Borjian A, Ralph B, et al. Impact of Leishmania mexicana infection on dendritic cell signaling and functions. PLoS Negl Trop Dis. 2014;8(9):e3202. doi: 10.1371/journal.pntd.0003202 25255446 PMC4177750

[52] Kropf P, Freudenberg MA, Modolell M, Price HP, Herath S, Antoniazi S, et al. Toll-like receptor 4 contributes to efficient control of infection with the protozoan parasite Leishmania major. Infect Immun. 2004;72(4):1920–8. doi: 10.1128/IAI.72.4.1920-1928.2004 15039311 PMC375159

[53] Liese J, Schleicher U, Bogdan C. TLR9 signaling is essential for the innate NK cell response in murine cutaneous leishmaniasis. Eur J Immunol. 2007;37(12):3424–34. doi: 10.1002/eji.200737182 18034422

[54] Ribeiro-Gomes FL, Moniz-de-Souza MC, Alexandre-Moreira MS, Dias WB, Lopes MF, Nunes MP, et al. Neutrophils activate macrophages for intracellular killing of Leishmania major through recruitment of TLR4 by neutrophil elastase. J Immunol. 2007;179(6):3988–94. doi: 10.4049/jimmunol.179.6.3988 17785837

[55] Ronet C, Passelli K, Charmoy M, Scarpellino L, Myburgh E, Hauyon La Torre Y, et al. TLR2 Signaling in Skin Nonhematopoietic Cells Induces Early Neutrophil Recruitment in Response to Leishmania major Infection. J Invest Dermatol. 2019;139(6):1318–28. doi: 10.1016/j.jid.2018.12.012 30594488 PMC8024985

[56] Regli IB, Passelli K, Martínez-Salazar B, Amore J, Hurrell BP, Müller AJ, et al. TLR7 Sensing by Neutrophils Is Critical for the Control of Cutaneous Leishmaniasis. Cell Reports. 2020;31(10):107746. doi: 10.1016/j.celrep.2020.107746 32521271

[57] Lima-Junior DS, Mineo TWP, Calich VLG, Zamboni DS. Dectin-1 Activation during Leishmania amazonensis Phagocytosis Prompts Syk-Dependent Reactive Oxygen Species Production To Trigger Inflammasome Assembly and Restriction of Parasite Replication. J Immunol. 2017;199(6):2055–68. doi: 10.4049/jimmunol.1700258 28784846

[58] Novais FO, Carvalho AM, Clark ML, Carvalho LP, Beiting DP, Brodsky IE, et al. CD8+ T cell cytotoxicity mediates pathology in the skin by inflammasome activation and IL-1β production. PLOS Pathogens. 2017;13(2):e1006196. 28192528 10.1371/journal.ppat.1006196 PMC5325592

[59] Lima-Junior DS, Costa DL, Carregaro V, Cunha LD, Silva ALN, Mineo TWP, et al. Inflammasome-derived IL-1β production induces nitric oxide–mediated resistance to Leishmania. Nature Medicine. 2013;19(7):909–15. 10.1038/nm.3221 23749230

[60] Barnett KC, Li S, Liang K, Ting JP. A 360° view of the inflammasome: Mechanisms of activation, cell death, and diseases. Cell. 2023;186(11):2288–312. 37236155 10.1016/j.cell.2023.04.025 PMC10228754

[61] Planès R, Pinilla M, Santoni K, Hessel A, Passemar C, Lay K, et al. Human NLRP1 is a sensor of pathogenic coronavirus 3CL proteases in lung epithelial cells. Molecular Cell. 2022;82(13):2385–400.e9. doi: 10.1016/j.molcel.2022.04.033 35594856 PMC9108100

[62] Tsu BV, Beierschmitt C, Ryan AP, Agarwal R, Mitchell PS, Daugherty MD. Diverse viral proteases activate the NLRP1 inflammasome. Elife. 2021;10. doi: 10.7554/eLife.60609 33410748 PMC7857732

[63] Robinson KS, Teo DET, Tan KS, Toh GA, Ong HH, Lim CK, et al. Enteroviral 3C protease activates the human NLRP1 inflammasome in airway epithelia. Science. 2020;370(6521). doi: 10.1126/science.aay2002 33093214

[64] Pinilla M, Mazars R, Vergé R, Gorse L, Paradis M, Suire B, et al. EEF2-inactivating toxins engage the NLRP1 inflammasome and promote epithelial barrier disruption. J Exp Med. 2023;220(10). doi: 10.1084/jem.20230104 37642996 PMC10465324

[65] Robinson KS, Toh GA, Firdaus MJ, Tham KC, Rozario P, Lim CK, et al. Diphtheria toxin activates ribotoxic stress and NLRP1 inflammasome-driven pyroptosis. J Exp Med. 2023;220(10). doi: 10.1084/jem.20230105 37642997 PMC10465786

[66] Jenster LM, Lange KE, Normann S, vom Hemdt A, Wuerth JD, Schiffelers LDJ, et al. P38 kinases mediate NLRP1 inflammasome activation after ribotoxic stress response and virus infection. J Exp Med. 2023;220(1). doi: 10.1084/jem.20220837 36315050 PMC9623368

[67] Robinson KS, Toh GA, Rozario P, Chua R, Bauernfried S, Sun Z, et al. ZAKα-driven ribotoxic stress response activates the human NLRP1 inflammasome. Science. 2022;377(6603):328–35. 35857590 10.1126/science.abl6324 PMC7614315

[68] Ewald SE, Chavarria-Smith J, Boothroyd JC. NLRP1 is an inflammasome sensor for Toxoplasma gondii. Infect Immun. 2014;82(1):460–8. doi: 10.1128/IAI.01170-13 24218483 PMC3911858

[69] Chavarría-Smith J, Vance RE. Direct Proteolytic Cleavage of NLRP1B Is Necessary and Sufficient for Inflammasome Activation by Anthrax Lethal Factor. PLOS Pathogens. 2013;9(6):e1003452. doi: 10.1371/journal.ppat.1003452 23818853 PMC3688554

[70] Levinsohn JL, Newman ZL, Hellmich KA, Fattah R, Getz MA, Liu S, et al. Anthrax lethal factor cleavage of Nlrp1 is required for activation of the inflammasome. PLoS Pathog. 2012;8(3):e1002638. doi: 10.1371/journal.ppat.1002638 22479187 PMC3315489

[71] Boyden ED, Dietrich WF. Nalp1b controls mouse macrophage susceptibility to anthrax lethal toxin. Nat Genet. 2006;38(2):240–4. doi: 10.1038/ng1724 16429160

[72] Calabrese L, Fiocco Z, Mellett M, Aoki R, Rubegni P, French LE, et al. The Role of NLRP1 Inflammasome in Skin Cancer and Inflammatory Skin Diseases. British Journal of Dermatology. 2023. 10.1093/bjd/ljad421 37889986

[73] Stojkov D, Claus MJ, Kozlowski E, Oberson K, Schären OP, Benarafa C, et al. NET formation is independent of gasdermin D and pyroptotic cell death. Sci Signal. 2023;16(769):eabm0517. doi: 10.1126/scisignal.abm0517 36693132

[74] Jorgensen I, Zhang Y, Krantz BA, Miao EA. Pyroptosis triggers pore-induced intracellular traps (PITs) that capture bacteria and lead to their clearance by efferocytosis. J Exp Med. 2016;213(10):2113–28. doi: 10.1084/jem.20151613 27573815 PMC5030797

[75] Ding J, Wang K, Liu W, She Y, Sun Q, Shi J, et al. Pore-forming activity and structural autoinhibition of the gasdermin family. Nature. 2016;535(7610):111–6. doi: 10.1038/nature18590 27281216

[76] Volkmar K, Jaedtka M, Baars I, Walber B, Philipp MS, Bagola K, et al. Investigating pyroptosis as a mechanism of L. major cell-to-cell spread in the human BLaER1 infection model. Mol Microbiol. 2024;121(3):453–69. doi: 10.1111/mmi.15142 37612878

[77] Rosazza T, Lecoeur H, Blisnick T, Moya-Nilges M, Pescher P, Bastin P, et al. Dynamic imaging reveals surface exposure of virulent Leishmania amastigotes during pyroptosis of infected macrophages. J Cell Sci. 2020;134(5). doi: 10.1242/jcs.242776 32501279

[78] Martinez-Rojano H, Mancilla-Ramirez J, Quiñonez-Diaz L, Galindo-Sevilla N. Activity of hydroxyurea against Leishmania mexicana. Antimicrob Agents Chemother. 2008;52(10):3642–7. doi: 10.1128/AAC.00124-08 18694950 PMC2565871

[79] Oh C, Li L, Verma A, Reuven AD, Miao EA, Bliska JB, et al. Neutrophil inflammasomes sense the subcellular delivery route of translocated bacterial effectors and toxins. Cell Rep. 2022;41(8):111688. doi: 10.1016/j.celrep.2022.111688 36417874 PMC9827617

[80] Masters Seth L, Gerlic M, Metcalf D, Preston S, Pellegrini M, O’Donnell Joanne A, et al. NLRP1 Inflammasome Activation Induces Pyroptosis of Hematopoietic Progenitor Cells. Immunity. 2012;37(6):1009–23. doi: 10.1016/j.immuni.2012.08.027 23219391 PMC4275304

[81] Schneider KS, Groß CJ, Dreier RF, Saller BS, Mishra R, Gorka O, et al. The Inflammasome Drives GSDMD-Independent Secondary Pyroptosis and IL-1 Release in the Absence of Caspase-1 Protease Activity. Cell Rep. 2017;21(13):3846–59. 29281832 10.1016/j.celrep.2017.12.018 PMC5750195

[82] Martinon F, Pétrilli V, Mayor A, Tardivel A, Tschopp J. Gout-associated uric acid crystals activate the NALP3 inflammasome. Nature. 2006;440(7081):237–41. doi: 10.1038/nature04516 16407889

[83] Mariathasan S, Newton K, Monack DM, Vucic D, French DM, Lee WP, et al. Differential activation of the inflammasome by caspase-1 adaptors ASC and Ipaf. Nature. 2004;430(6996):213–8. doi: 10.1038/nature02664 15190255

[84] Hermann M, Stillhard P, Wildner H, Seruggia D, Kapp V, Sánchez-Iranzo H, et al. Binary recombinase systems for high-resolution conditional mutagenesis. Nucleic Acids Res. 2014;42(6):3894–907. doi: 10.1093/nar/gkt1361 24413561 PMC3973285

[85] Sörensen M, Lippuner C, Kaiser T, Mißlitz A, Aebischer T, Bumann D. Rapidly maturing red fluorescent protein variants with strongly enhanced brightness in bacteria. FEBS Letters. 2003;552(2–3):110–4. doi: 10.1016/s0014-5793(03)00856-1 14527670

[86] Neva FA, Wyler D, Nash T. Cutaneous leishmaniasis—a case with persistent organisms after treatment in presence of normal immune response. Am J Trop Med Hyg. 1979;28(3):467–71. 222157

[87] Späth GF, Beverley SM. A lipophosphoglycan-independent method for isolation of infective Leishmania metacyclic promastigotes by density gradient centrifugation. Exp Parasitol. 2001;99(2):97–103. doi: 10.1006/expr.2001.4656 11748963

[88] DeSouza-Vieira T, Iniguez E, Serafim TD, de Castro W, Karmakar S, Disotuar MM, et al. Heme Oxygenase-1 Induction by Blood-Feeding Arthropods Controls Skin Inflammation and Promotes Disease Tolerance. Cell Rep. 2020;33(4):108317. doi: 10.1016/j.celrep.2020.108317 33113362

[89] Díaz-Varela M, Sanchez-Hidalgo A, Calderon-Copete S, Tacchini V, Shipley TR, Ramírez LG, et al. The different impact of drug-resistant Leishmania on the transcription programs activated in neutrophils. iScience. 2024;27(5):109773. doi: 10.1016/j.isci.2024.109773 38711445 PMC11070714

