# Supplementary figures and images for: NLRP1-dependent activation of Gasdermin D in neutrophils controls cutaneous leishmaniasis
Source: PLoS Pathog. 2024 Sep 9;20(9):e1012527. doi: 10.1371/journal.ppat.1012527 (PMC11412672; doi:10.1371/journal.ppat.1012527)

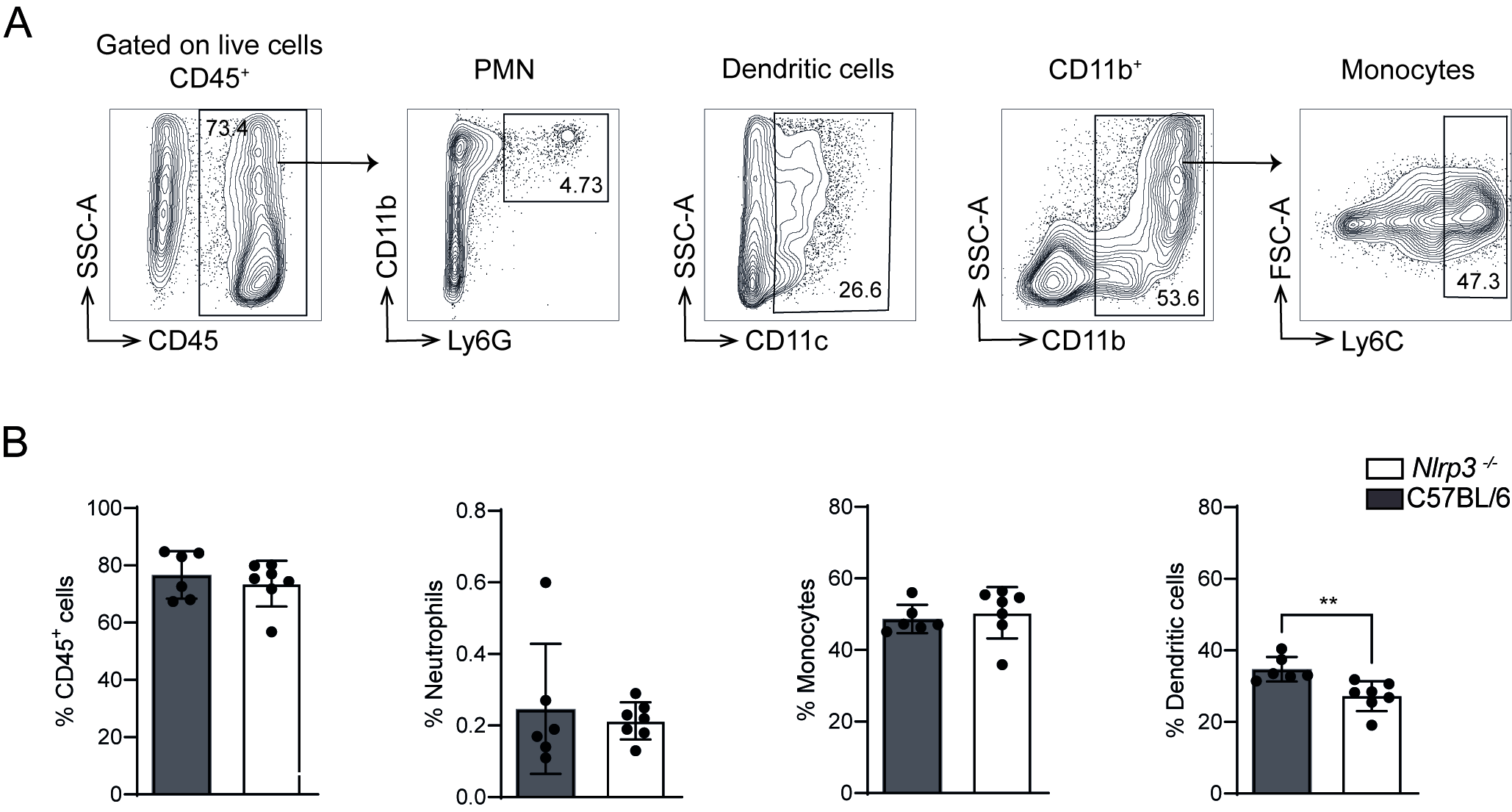

Supplement: S1 Fig — Nlrp3-/- and C57BL/6 mice were infected i.d with 106 L. mexicana promastigotes and lesion development was measured over time. (A) General flow cytometry gating strategy for infected ears. (B) Corresponding frequencies of ear CD45+ cells, CD45+CD11b+Ly6G+ neutrophils, CD45+CD11b+Ly6C+ monocytes, and CD45+CD11C+ dendritic cells. Data are shown as mean ± SD and statistical differences were analyzed with a Mann-Whitney U test. Data are representative of ≥3 experiments with n≥4/group. ***p <0.001 (TIF) [file ppat.1012527.s001.tif]

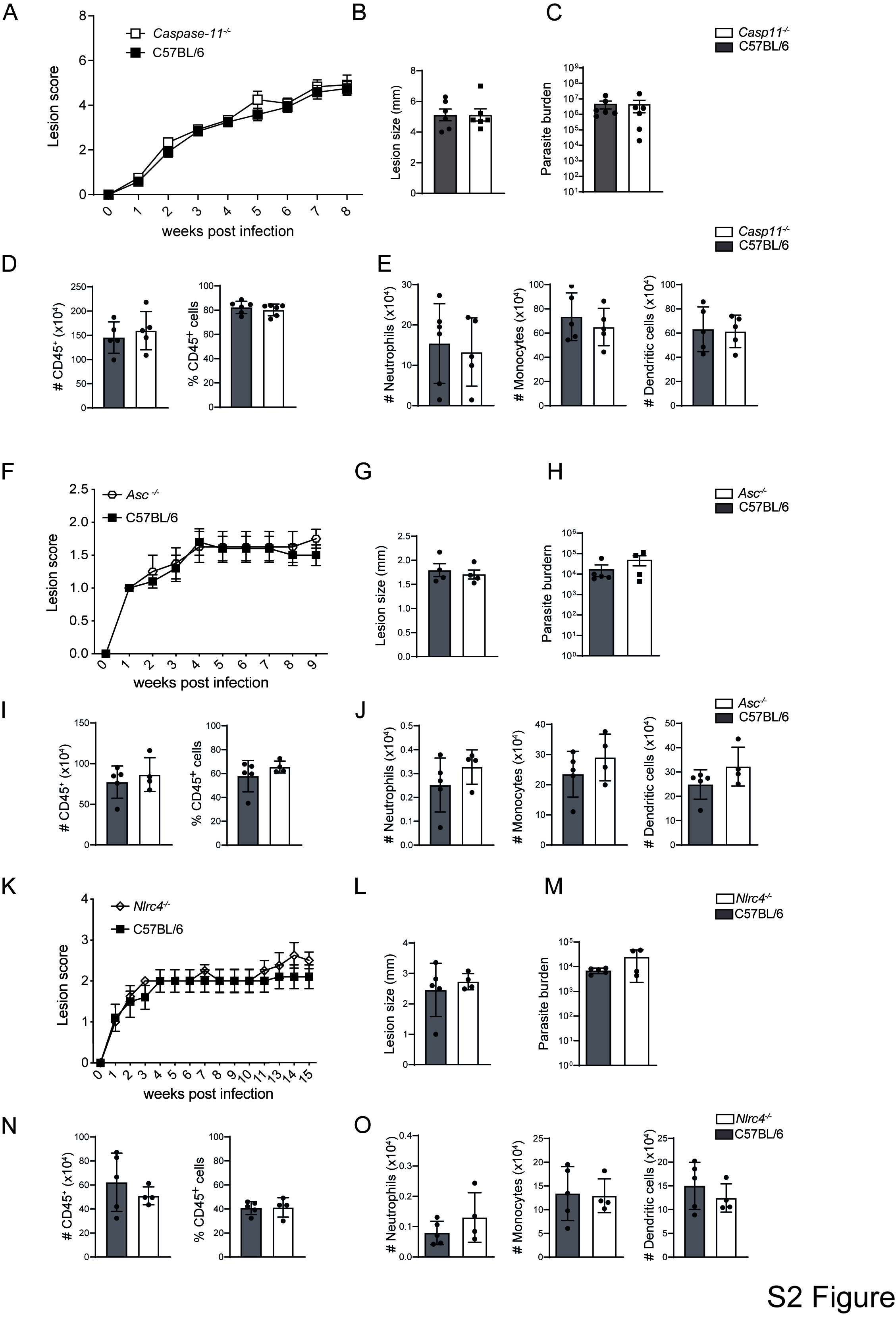

Supplement: S2 Fig — (A) Casp11-/- and C57BL/6 mice were infected with L. mexicana promastigotes and lesion score was measured over time. (B) Lesion size at 9 weeks p.i. and (C) parasite load at the site of infection as determined by LDA. (D) The number and frequency of CD45+ cells were analyzed by flow cytometry. (E) The number of CD45+CD11b+Ly6G+ neutrophils, CD45+CD11b+Ly6C+ monocytes, and CD45+CD11C+ dendritic cells, as determined by flow cytometry. (F) Asc-/-and C57BL/6 control mice were infected with L. mexicana promastigotes and lesion score was measured over time. (G) Lesion size and (H) parasite burden at 9 weeks p.i. (I) The number and frequency of CD45+ cells and (J) the number of CD45+CD11b+Ly6G+ neutrophils, CD45+CD11b+Ly6C+ monocytes, and CD45+CD11C+ dendritic cells at the site of infection. (K) Nlrc4-/- and C57BL/6 control mice were similarly infected with L. mexicana and lesion score was measured over the course of infection. (L) Fifteen weeks p.i., the lesion size was measured, with parasite burden (M), determined by LDA, and (N) the number and frequency of dermal CD45+ cells were determined by flow cytometry. (O) The numbers of CD45+CD11b+Ly6G+ neutrophils, CD45+CD11b+Ly6C+ monocytes, and CD45+CD11C+ dendritic cells were analyzed by flow cytometry. Data are shown as mean ± SD and statistical differences in lesion development were analyzed with a 2-way ANOVA. Data are representative of ≥3 experiments with n≥4/group. (TIF) [file ppat.1012527.s002.tif]

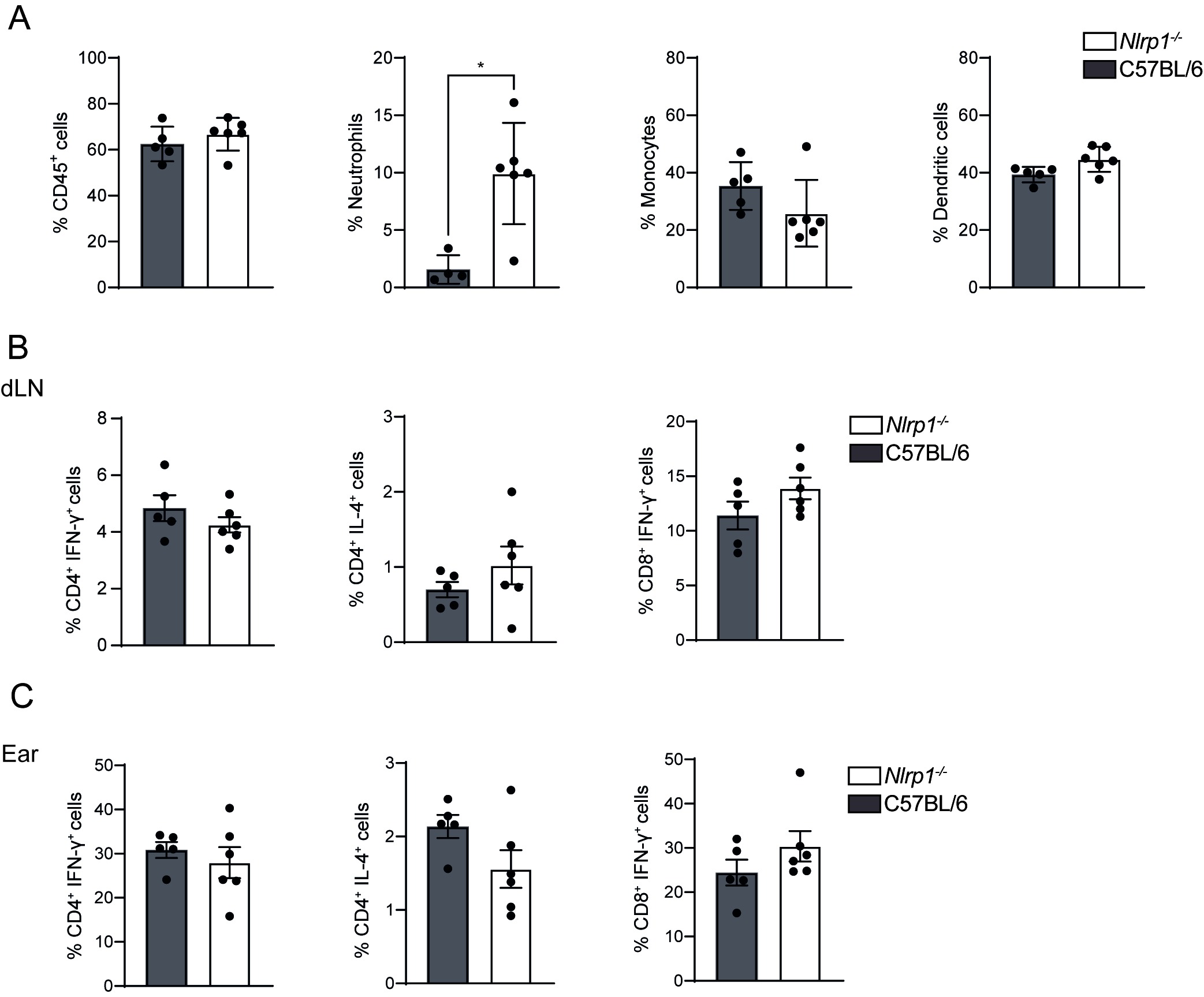

Supplement: S3 Fig — (A) Nlrp1-/- and C57BL/6 control mice were infected i.d. with metacyclic L. mexicana promastigotes and 8 weeks p.i., and the frequency of CD45+CD11b+Ly6G+ neutrophils, CD45+CD11b+Ly6C+ monocytes, and CD45+CD11c+ dendritic cells in infected ears was analyzed by flow cytometry. (B) The frequency of CD4+ IFN-γ+, CD4+ IL-4+, and CD8+ IFN-γ+ T cells in draining lymph nodes (dLN) and (C) ears was analyzed by flow cytometry. (TIF) [file ppat.1012527.s003.tif]

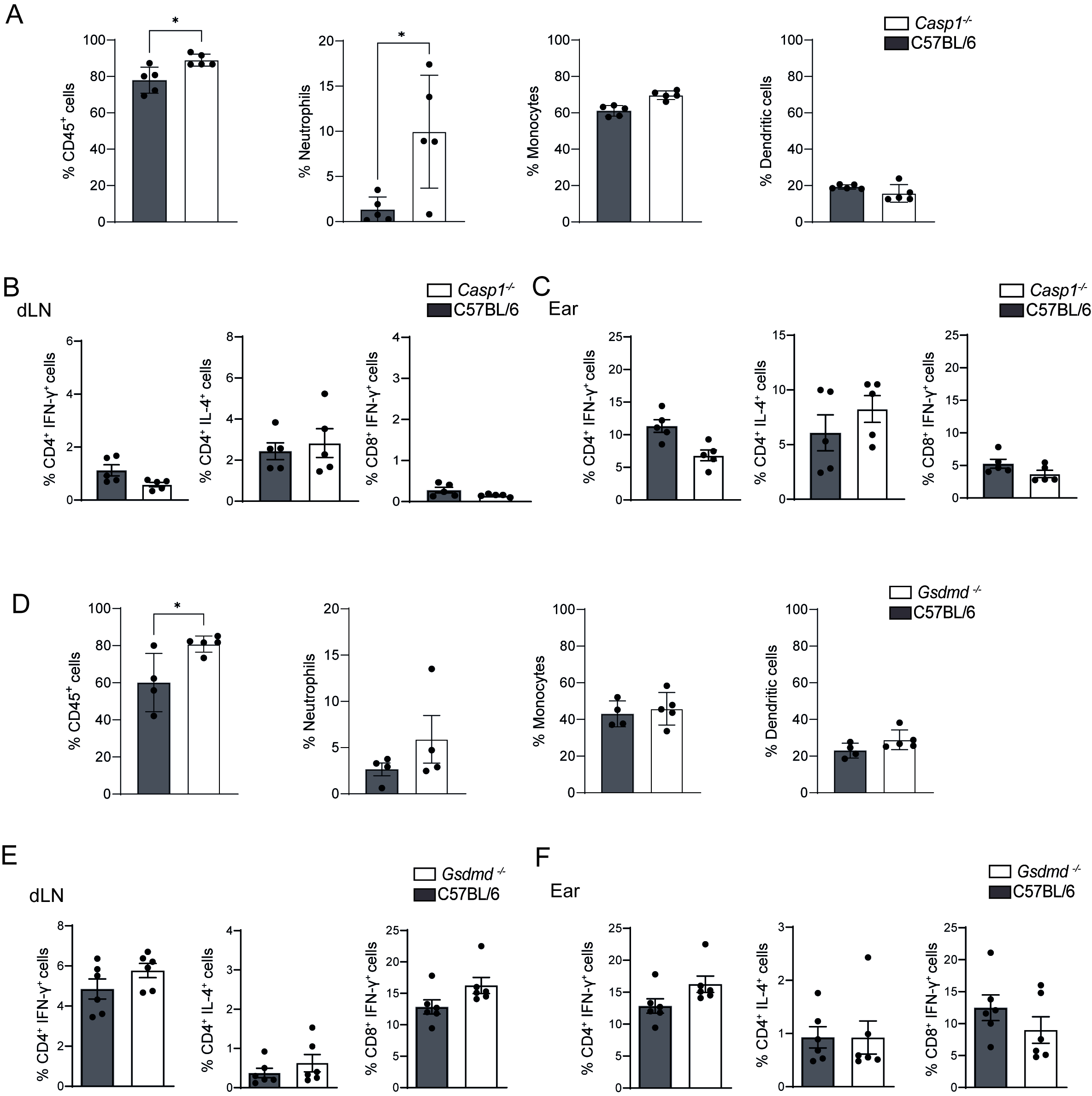

Supplement: S4 Fig — (A) Casp1-/- and C57BL/6 control mice were infected i.d. with metacyclic L. mexicana promastigotes and 7 weeks p.i. the frequency of CD45+CD11b+Ly6G+ neutrophils, CD45+CD11b+Ly6C+ monocytes, and CD45+CD11c+ dendritic cells in infected ears was analyzed by flow cytometry. (B) The frequency of CD4+ IFN-γ+, CD4+ IL-4+, and CD8+ IFN-γ+ T cells present in dLN and (C) infected ears was analyzed by flow cytometry. (D) Gsdmd-/- and C57BL/6 control mice were similarly infected for 8 weeks and the frequency of CD45+CD11b+Ly6G+ neutrophils, CD45+CD11b+Ly6C+ monocytes, and CD45+CD11c+ dendritic cells in infected ears was determined by flow cytometry. (E) The frequency of CD4+ IFN-γ+, CD4+ IL-4+, and CD8+ IFN-γ+ T cells in dLN and (F) ear was similarly analyzed by flow cytometry. Data are shown as mean ± SD and statistical differences between groups were analyzed by Mann-Whitney U-test and are representative of ≥3 experiments with n≥4/group. *p <0.05. (TIF) [file ppat.1012527.s004.tif]

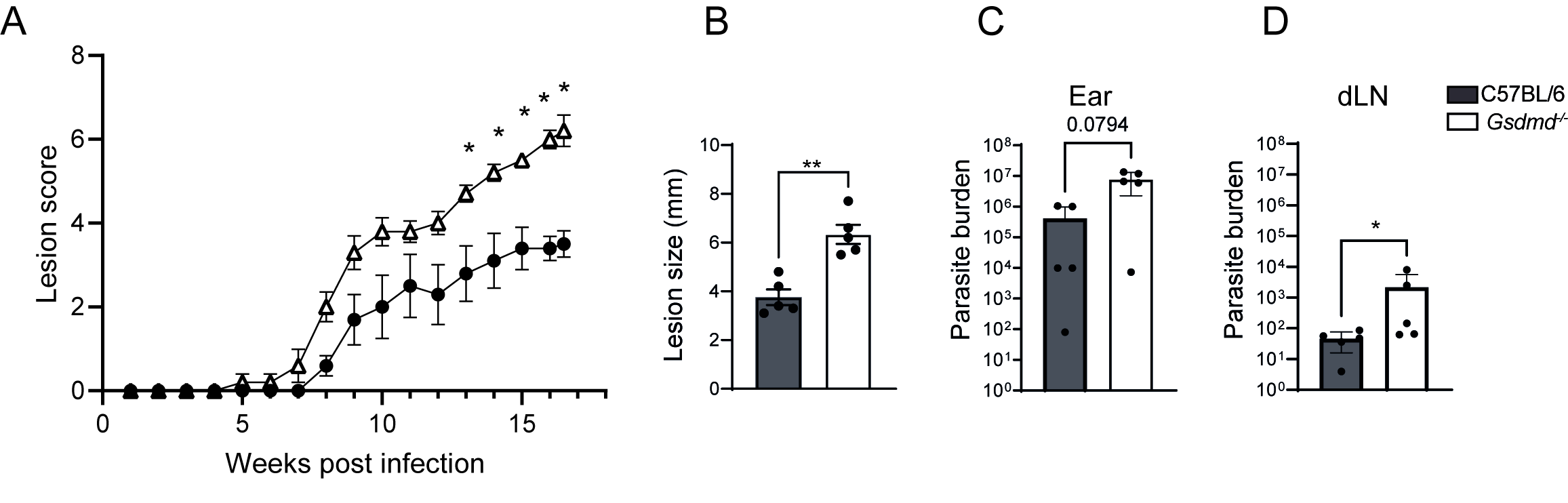

Supplement: S5 Fig — (A) Gsdmd-/-and C57BL/6 were infected i.d. with a low dose (104) of L. mexicana and lesion progression was assessed over 16 weeks. (B) Lesion size and parasite burden at 16 weeks p.i., at the infection site (C) and in the dLN (D). Data are shown as mean ± SEM and are representative of ≥2 experiments, n≥4/group. Statistical differences in lesion development were analyzed with a 2-way ANOVA with repeated measures, and parasite number and cell percentages using a Mann-Whitney U-test. *p <0.05; **p <0.01 (TIF) [file ppat.1012527.s005.tif]

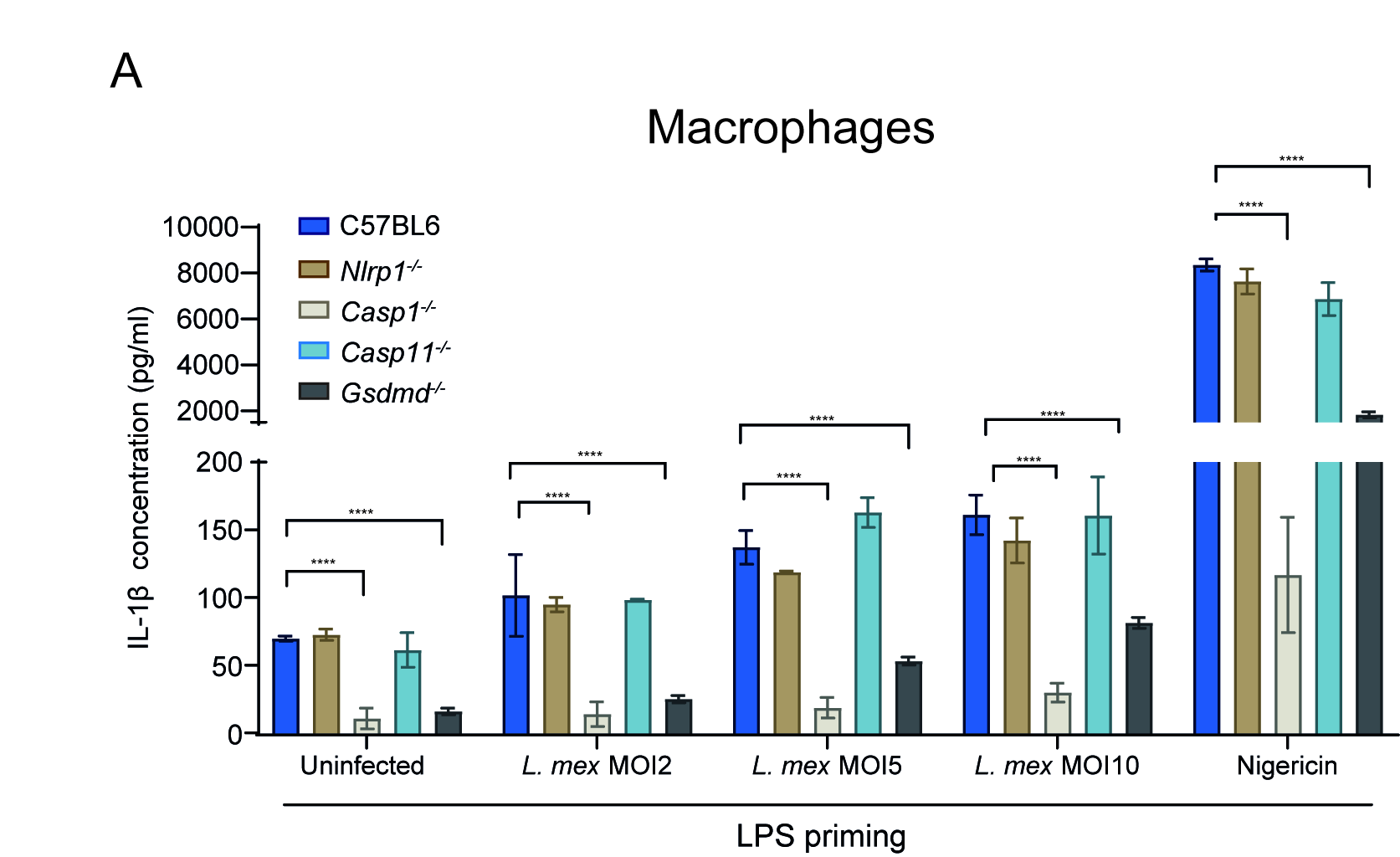

Supplement: S6 Fig — (A) LPS-primed, C57BL/6, Nlrp1-/-, Casp1-/-, Casp11-/ and Gsdmd-/- bone marrow-derived macrophages (BMDMs) were infected with L. mexicana promastigotes at an MOI of 2, 5 and 10 for 16h, or exposed to nigericin for 4 hours, as a positive control. IL-1β concentration was measured in cell supernatants by ELISA. Data are shown as mean ± SD and are representative of n>3 experiments. 2-way ANOVA with Dunnett’s multiple comparison, *p <0.05; **p <0.01, ***p>0001. (TIF) [file ppat.1012527.s006.tif]

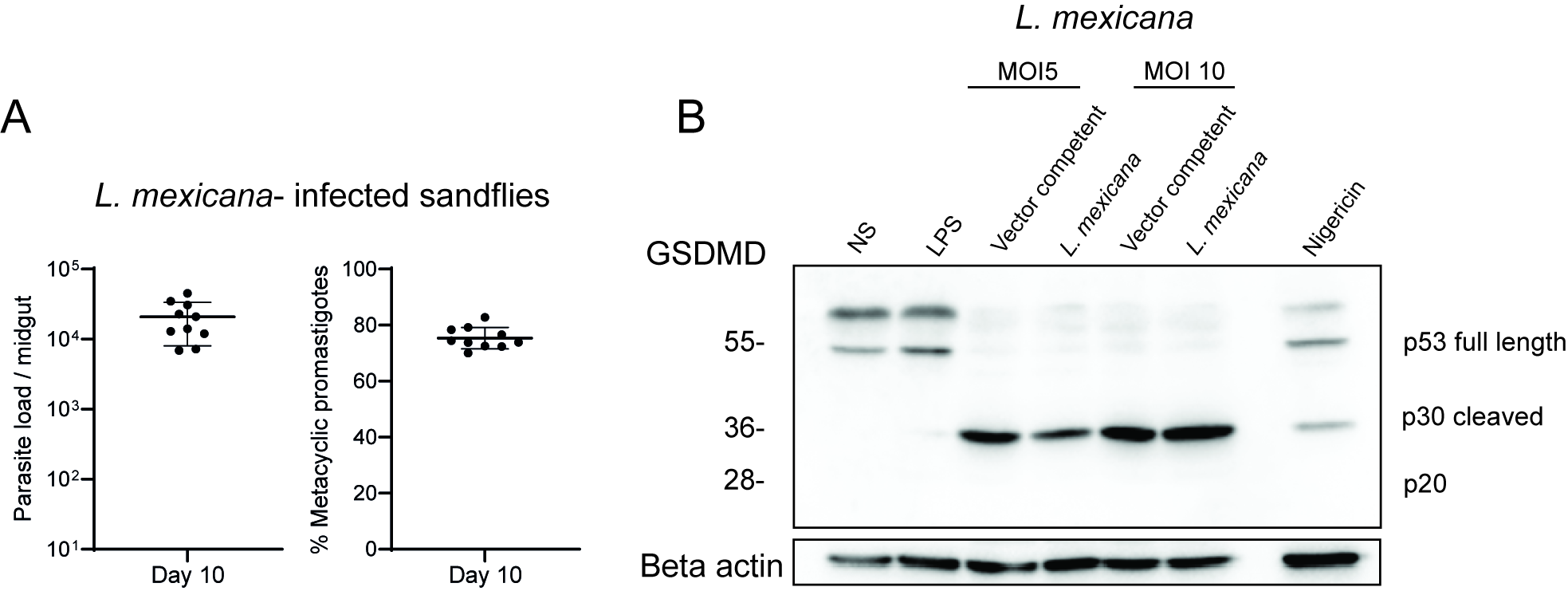

Supplement: S7 Fig — (A) To assess vector competency of Leishmania mexicana in Lutzomyia longipalpis, sand flies were infected with 5x10^6^/ mL procyclic promastigote parasites. Infection was assessed by dissecting 5–10 sand fly midguts at day 10 post-infection. The total number of parasites and the percentage of infectious metacyclic promastigotes per midgut were counted. Data are representative of two independent experiments and are represented as mean ± SD. (B) LPS-primed C57BL/6 BMNs were infected with vector-competent L. mexicana metacyclic promastigotes or the same strain maintained in standard growing media and passaged in BALB/c mice. BMNs were infected for 16 hours at MOI 5 or 10 and cell lysates were subjected to immunoblotting for GSDMD cleavage and β-actin. Nigericin was used as a positive control. Data are representative for n≥2 independent experiments. (TIF) [file ppat.1012527.s007.tif]

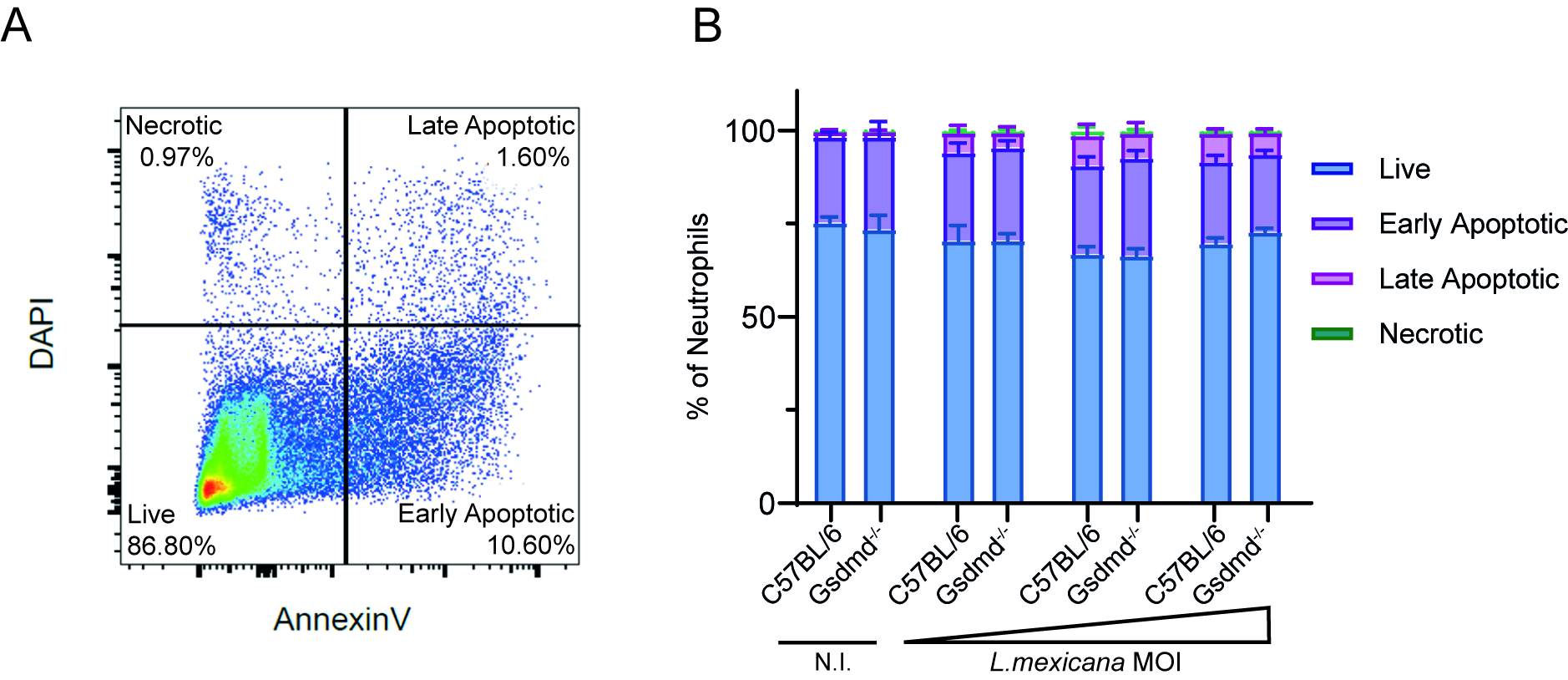

Supplement: S8 Fig — (A) LPS-primed BMNs of C57BL/6 and Gsdmd-/- were infected at an MOI of 2, 5, and 10, and 16 hours later, neutrophils were stained with Annexin-V and DAPI to assess apoptosis by flow cytometry. A representative plot is shown and (B) the relative frequency of viable, early apoptotic, late apoptotic, and necrotic cells is given. Data are shown as mean ± SD and are representative of ≥2 experiments. (TIF) [file ppat.1012527.s008.tif]

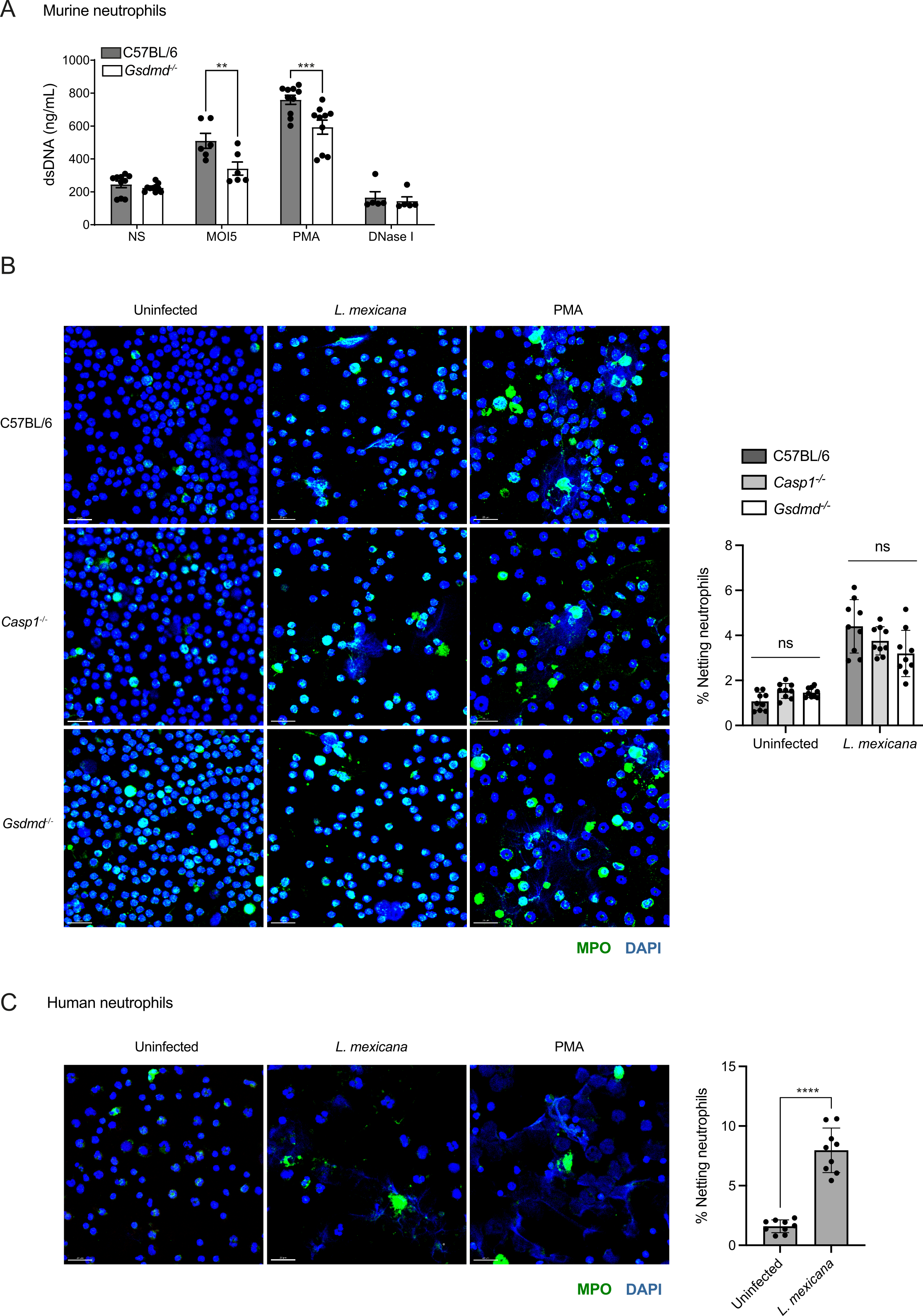

Supplement: S9 Fig — (A) BMNs were isolated and infected with L. mexicana at an MOI5. Four hours later, dsDNA release was analyzed by Picogreen assay. PMA was used as a positive control and BMNs treated with DNAse I as a negative control. Results are shown as a pool of 3 replicates. n = 3/group. (B) Ex vivo NET formation of BMNs exposed for 4 hours to medium (uninfected), L. mexicana (MOI5) and PMA. Samples were fixed, stained for MPO and DNA (DAPI) and analyzed by confocal microscopy. Representative confocal microscopy images are shown. On the right, the frequency of netting neutrophils is shown, with NETs defined by colocalization of decondensed DNA and MPO. n = 9 random fields/group. (C) Ex vivo NET formation of isolated human neutrophils exposed for 4 hours to medium, L. mexicana (MOI5), or PMA. Representative pictures and frequency of netting neutrophils is shown. n = 9 random fields/group. Scale bar: 20 μm. Data are shown as mean ± SD. One representative experiment out of two is shown in each graph (B, C). *p <0.05, **p <0.01, ***p <0.001, ns: non-significant, as determined by Kruskal-Wallis (B) and Mann-Whitney U-test (A, C). (TIFF) [file ppat.1012527.s009.tiff]

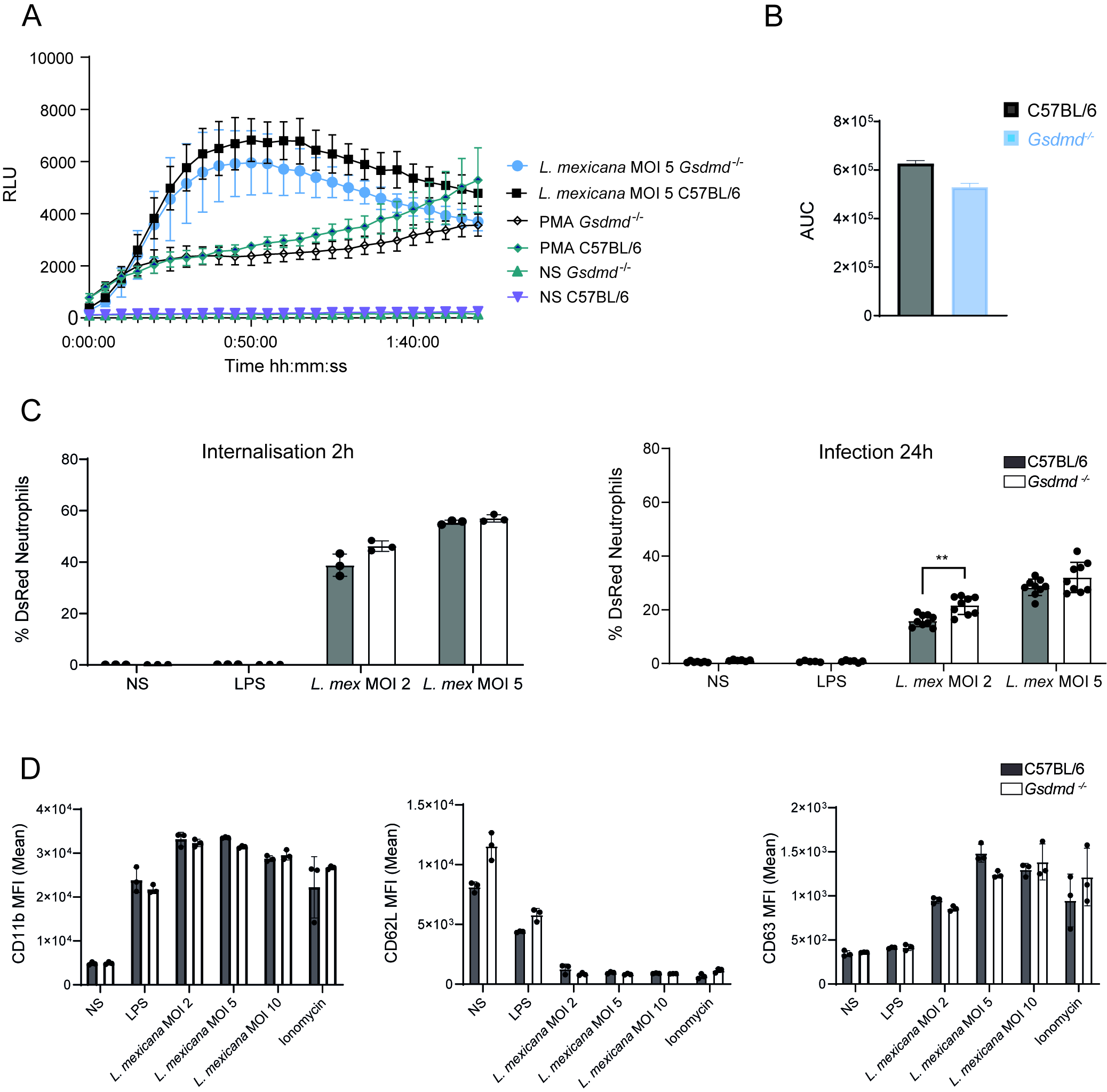

Supplement: S10 Fig — (A) BMNs of Gsdmd-/- and C57BL/6 mice were infected with L. mexicana promastigotes at MOI 5 and reactive oxygen species (ROS) production was measured over 2h by the addition of luminol. Values are represented as mean relative light units (RLU) ± SD over time. PMA was used as a positive control, with non-stimulated (NS) as a negative control. (B) Area under the curve (AUC) ± SD of representative curves. Data are shown as mean ± SD and representative of ≥3 experiments. (C) LPS-primed Gsdmd-/- and C57BL/6 BMNs were infected with dsRed+-expressing L. mexicana promastigotes at the indicated MOI and the frequency of L. mexicana-dsRed+ neutrophils was analyzed by flow cytometry, at 2- and 24-hours p.i. respectively. (D) Neutrophil activation status and degranulation was analyzed by flow cytometry, showing the mean fluorescence intensity (MFI) of CD11b, CD62L, and CD63. Ionomycin was used as a positive control. Data are represented as mean ± SD and are representative of ≥2 experiments. NS (non-stimulated). 2-way ANOVA with Dunnett’s multiple comparison *p <0.05; **p <0.01. (TIF) [file ppat.1012527.s010.tif]

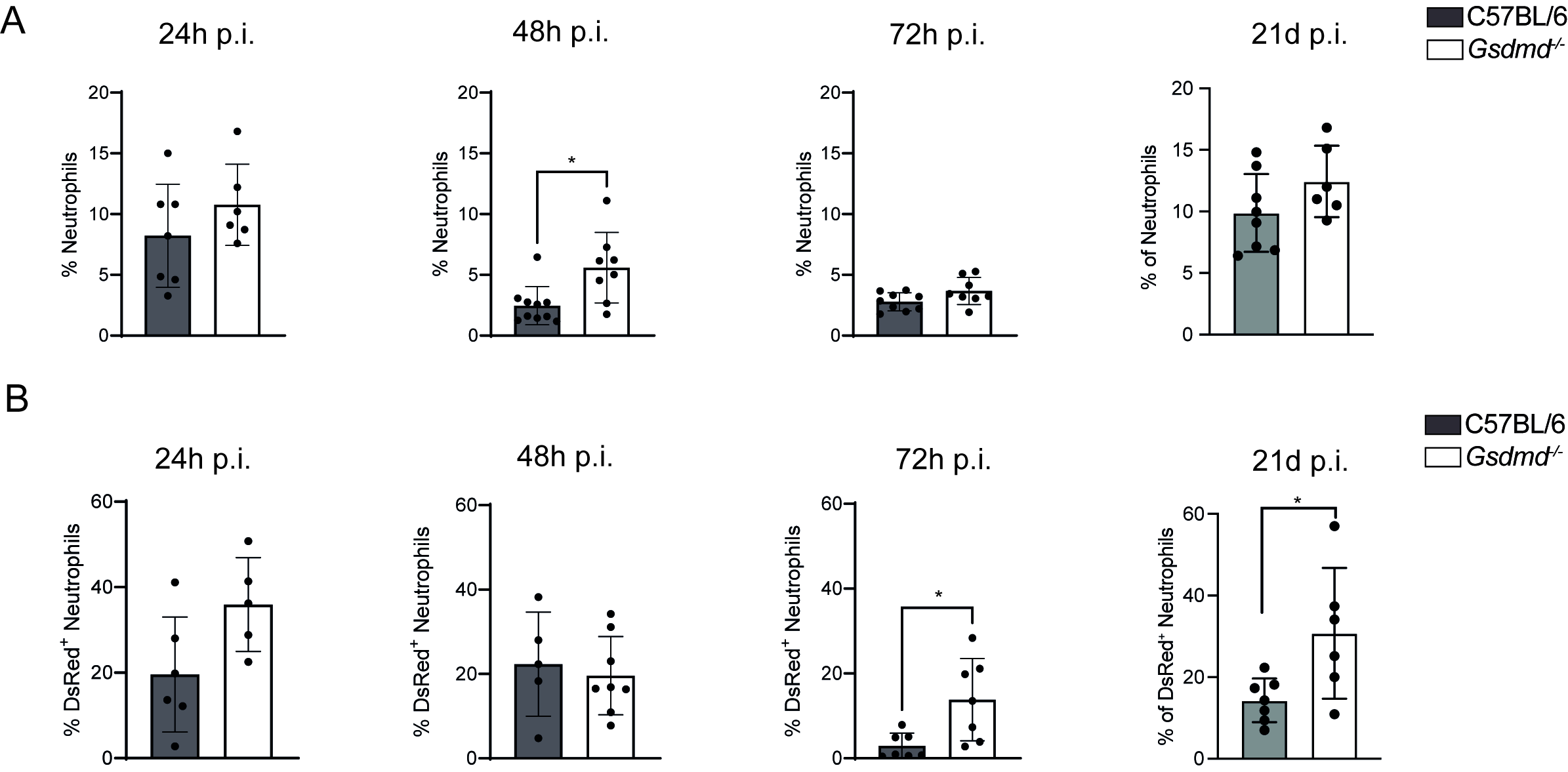

Supplement: S11 Fig — (A) C57BL/6 and Gsdmd-/- mice were infected i.d with metacyclic dsRed+ L. mexicana promastigotes and frequency of CD45+CD11b+Ly6G+ neutrophils was determined by flow cytometry at the indicated timepoints. (B) Frequency of infected CD45+CD11b+Ly6G+dsRed+ neutrophils at 24, 48, 72 hours and 21 days p.i. Data is representative of n≥2 independent experiments and differences were analyzed by Mann-Whitney U-test. *p <0.05. (TIF) [file ppat.1012527.s011.tif]

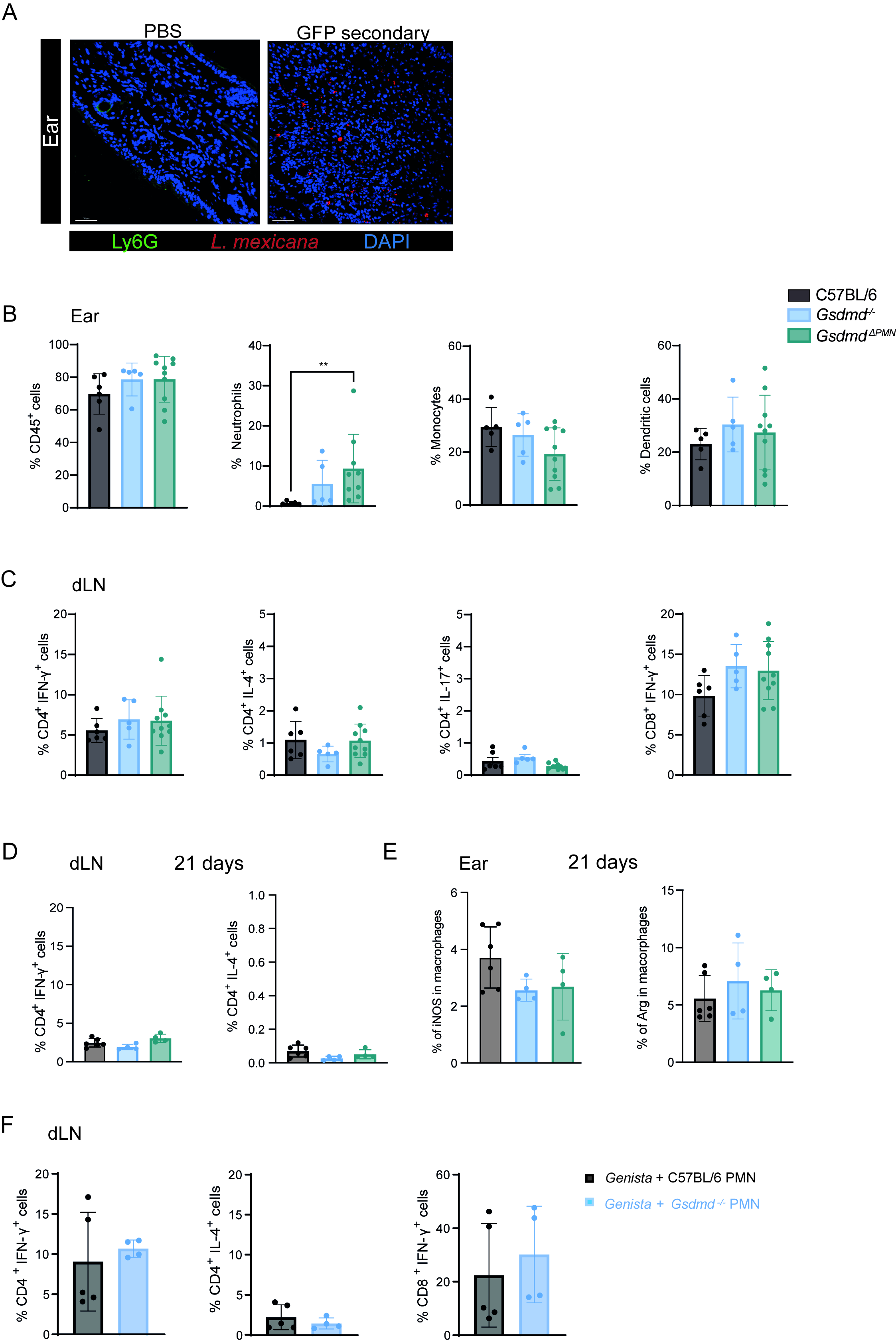

Supplement: S12 Fig — (A) Left panel: representative immunohistology pictures of PBS-injected ear stained for Ly6G+ neutrophils (green), and DAPI (blue), serving as technical negative control for DsRed. Right panel: technical negative control for anti-Ly6G antibody, showing the absence of Ly6G+ neutrophils (green) upon omission of primary antibody, DAPI (grey), and dsRed+ parasites. Scale bar, 50 μm. (B) Gsdmd-/-, GsdmdΔPMN, and WT, mice were infected i.d. with L. mexicana promastigotes. The frequency of CD45+ cells, CD45+CD11b+Ly6G+ neutrophils, CD45+CD11b+Ly6C+ monocytes, and CD45+CD11c+ dendritic cells at 11 weeks p.i. was analyzed by flow cytometry. (C) The frequency of CD4+ IFN-γ+, CD4+ IL-4+, CD4+ IL-17+ and CD8+ IFN-γ+ T cells in draining lymph nodes (dLN) was analyzed by flow cytometry at 11 weeks p.i., and (D) Mice were similarly infected and 21 days later, the frequency of CD4+ IFN-γ+ and CD4+ IL-4+ T cells was analyzed by flow cytometry (E) The frequency of arginase (Arg+) and iNOS positive CD45+CD11b+F480+ macrophages, as determined by flow cytometry. (F) C57BL/6 and Gsdmd-/- BMNs were isolated and adoptively transferred at the time of infection in two separate groups of Genista mice. The frequency of CD4+ IFN-γ+, CD4+ IL-4+ and CD8+ IFN-γ+ T cells after 10 weeks p.i. Data are representative of n≥2 independent experiments. Differences in cell populations were analyzed with Kruskall-Wallis with Dunn’s multiple comparisons test (B, C, D, E) or Mann-Whitney U test (D). **p <0.01. (TIF) [file ppat.1012527.s012.tif]
